# Treatment Patterns, Treatment Response, and Survival Outcomes in Patients with Multiple Myeloma: A Single-Center Real-World Study

**DOI:** 10.3390/jcm15218340

**Published:** 2026-10-26

**Authors:** Meryem Şener, Emel Gürkan, Mehmet Ali Uçar

**Affiliations:** Department of Hematology, Faculty of Medicine, Çukurova University, Adana 01330, Türkiye

**Keywords:** multiple myeloma, prognostic factors, treatment response

## Abstract

**Background/Objectives:** Real-world data on multiple myeloma (MM) treatment patterns under evolving drug access are limited. We describe first-line treatment over two decades and factors associated with survival. **Methods:** We retrospectively analyzed 254 patients diagnosed between 2002 and 2024. Survival was estimated with the Kaplan–Meier method; multivariable Cox models included prespecified covariates and autologous stem cell transplantation (ASCT) as a time-dependent covariate, non-proportional effects were estimated as interval-specific hazard ratios (HRs), and first-line response was assessed in 6-month landmark analyses. **Results:** Median age was 68 years. First-line induction shifted from VAD (64.9% until 2014) to VCD (44.0% in 2015–2019) and VRD (45.1% in 2020–2024). Transplantation was performed in 79 patients (31.1%), 58 as first-line consolidation. With a median follow-up of 101.2 months, median overall survival (OS) was 54.6 months and time to next line of therapy or death (TNLT) was 11.4 months. ISS stage III (HR 1.98) and elevated LDH (HR 1.55) were associated with shorter OS. The associations of ASCT and induction regimen with OS changed over follow-up: ASCT was associated with lower mortality during the first 5 years after diagnosis (HR 0.18 for 0–24 months and 0.20 for 24–60 months) but not thereafter (HR 0.99), and VD induction, compared with VCD, with higher mortality during the same period (HR 4.16 and 3.90). Stable or progressive disease after induction was associated with shorter OS (HR 2.93) and shorter TNLT (HR 3.55). Laboratory ratios were not prognostic. **Conclusions:** ISS stage, LDH, induction regimen, transplantation and early response were associated with survival in this real-world cohort. Time-dependent modeling of ASCT removes immortal-time bias but not selection by age, fitness, organ function and response, and treatment eras changed; these associations should therefore not be interpreted as treatment effects.

## 1. Introduction

Multiple myeloma (MM) is a clonal plasma-cell neoplasm characterized by the uncontrolled proliferation of malignant plasma cells within the bone marrow, and it is the second most common hematologic malignancy after non-Hodgkin lymphoma [1,2]. Its global incidence has risen steadily over the past three decades, with the largest relative increases reported in middle- and low-middle-income regions, and it remains a disease predominantly of older adults, with a median age at diagnosis in the late sixties [3]. Despite substantial improvements in survival over the past two decades, MM remains largely incurable, and its clinical course is markedly heterogeneous, ranging from indolent disease controlled for many years to aggressive, treatment-refractory presentations associated with early mortality [1,2].

The treatment landscape of MM has been transformed by proteasome inhibitors (e.g., bortezomib) and immunomodulatory drugs (e.g., thalidomide and lenalidomide), which replaced anthracycline-based regimens such as vincristine–doxorubicin–dexamethasone (VAD) and are combined in frontline induction regimens such as bortezomib–cyclophosphamide–dexamethasone (VCD) and bortezomib–lenalidomide–dexamethasone (VRD). More recently, anti-CD38 monoclonal antibodies have been incorporated into first-line therapy for both transplant-ineligible and transplant-eligible patients, with further gains in progression-free survival [4,5]. For transplant-eligible patients, high-dose therapy followed by autologous stem cell transplantation (ASCT) as consolidation after induction remains a cornerstone of first-line management, a strategy first validated in a landmark randomized trial and subsequently supported by meta-analytic evidence in the era of novel agents [6,7]. The availability of these agents has, however, differed considerably between countries and over time, so the regimens actually used in routine practice often lag behind those tested in clinical trials. Real-world data that describe how practice has changed, and how patients treated outside trials fare, are therefore needed [7].

Response to induction also carries prognostic information in clinical trials [8], but outcome in routine practice depends equally on baseline risk. Prognostic staging systems applied at diagnosis have been refined: the International Staging System (ISS), based on serum β2-microglobulin and albumin, provides a simple three-tier stratification, and the Revised ISS (R-ISS) adds high-risk cytogenetic abnormalities and serum lactate dehydrogenase (LDH) [9,10]. Together with the older Durie–Salmon system, these tools form the basis of baseline risk assessment in everyday practice [11].

In addition to formal staging systems, routinely available laboratory parameters, including serum immunoglobulins, serum free light chains, and inflammation-based indices such as the neutrophil-to-lymphocyte ratio (NLR) and platelet-to-lymphocyte ratio (PLR), have been proposed as low-cost prognostic markers in MM. The evidence for their independent value is inconsistent: a recent meta-analysis reported an association between elevated NLR and inferior survival but not for PLR, with considerable heterogeneity in cut-offs and populations [12].

Much of the evidence underlying current staging systems and treatment algorithms has been generated in clinical trial populations or large registries, which may not capture the treatment patterns, comorbidity burden and outcomes of routine practice. Single-center real-world studies, such as those from Japan and from cohorts with extramedullary disease, continue to provide complementary evidence [13,14], but comparable long-term data from Türkiye, where access to novel agents has changed substantially over the last two decades, are scarce. Real-world cohorts include the older patients and those with renal impairment or comorbidity who are often excluded from trials, and they show how treatment sequences, including the use of transplantation, follow drug availability and reimbursement rather than trial protocols. Such data are needed to interpret trial results in routine practice and to plan the allocation of new agents.

The primary objective of this study was to describe first-line treatment patterns, including the use of ASCT, and their evolution over time in patients with MM treated at a tertiary hematology center over a 22-year period. The secondary objectives were to estimate overall survival and time to next line of therapy or death and to identify baseline and treatment-related factors associated with these outcomes, including the prognostic value of established staging systems, response to first-line induction and routinely measured laboratory parameters.

## 2. Materials and Methods

### 2.1. Study Design and Patients

This single-center, retrospective, non-interventional cohort study was conducted in accordance with the principles of the Declaration of Helsinki and was approved by the Çukurova University Faculty of Medicine Research Ethics Committee (Meeting No. 146; Decision No. 34; approval date: 26 July 2024). Owing to the retrospective design and the use of de-identified, pre-existing medical records, the requirement for individual informed consent was waived by the ethics committee.

All patients with MM who were diagnosed and treated at the Department of Hematology, Çukurova University Faculty of Medicine, and whose records were available in the departmental database were retrospectively screened. The included patients were diagnosed between May 2002 and May 2024 (232 patients [91.3%] from 2010 onward), and follow-up data were extracted up to an administrative cut-off of 31 December 2025. The diagnosis of MM was established according to the International Myeloma Working Group (IMWG) diagnostic criteria [15]. All 254 consecutive patients meeting these criteria were included, and no patient was excluded; patients with missing data for individual variables were retained, and the number of patients with available data is reported for each variable.

### 2.2. Data Collection and Definitions

Demographic data, disease characteristics and laboratory parameters were retrieved from electronic and paper-based medical records. Laboratory values and disease stage refer to the time of diagnosis. Comorbidities were extracted from the medical records and grouped as hypertension, diabetes, cardiac, chronic kidney, chronic lung and cerebrovascular or neurological disease, other malignancy and other conditions; conditions related to the plasma-cell disorder (plasmacytoma, amyloidosis and POEMS syndrome) were not counted as comorbidities. Renal impairment was defined as serum creatinine ≥ 2 mg/dL. Eastern Cooperative Oncology Group (ECOG) performance status was not recorded consistently in the database and could not be analyzed. Disease stage was classified according to the Durie–Salmon system, the ISS and the R-ISS [9,10,11].

Cytogenetic risk was assessed by interphase fluorescence in situ hybridization (FISH) on bone-marrow plasma cells for TP53/del(17p), t(4; 14), t(11; 14) and del(13q14); t(14; 16) was not part of the institutional panel, so some high-risk patients may have been classified as standard risk. High-risk cytogenetics was defined as del(17p) and/or t(4; 14). The R-ISS was calculated from ISS stage, serum LDH (upper limit of normal, 240 U/L) and FISH-defined high-risk cytogenetics. Patients with ISS stage II were assigned R-ISS II irrespective of the other components; patients with ISS stage I or III were assigned an R-ISS stage only when the components required to distinguish R-ISS I from II, or II from III, were available. Under these rules, R-ISS II for ISS II, R-ISS II for ISS I with elevated LDH and R-ISS III for ISS III with elevated LDH could be assigned without cytogenetic data, and R-ISS III for ISS III with normal LDH required high-risk FISH. R-ISS I, and R-ISS II for ISS III with normal LDH, required standard-risk FISH and therefore assumed the absence of the untested t(14; 16); these assignments are reported separately as modified R-ISS, and untested patients were never assumed to have standard-risk cytogenetics. ISS, which was available for 241 patients, was used as the staging variable in the primary multivariable models.

Lines of therapy were defined according to IMWG consensus principles [16]: a planned sequence of induction, consolidation with ASCT and maintenance constitutes one line, and a new line starts when the planned therapy is changed because of progression, relapse or toxicity. Because the reason for each change of therapy was not recorded systematically in the database, changes were classified with prespecified rules from the recorded sequence, dates and best response of each regimen. High-dose melphalan with ASCT, and lenalidomide or thalidomide maintenance started within 12 months of transplantation, were considered part of the line in which they were given. A change of induction regimen within 6 months of the start of the previous regimen in a patient who had achieved at least a minimal response (MR) was regarded as an induction modification for suboptimal response within the first line; this mainly concerned patients in PR after VAD or VCD who were switched to another proteasome inhibitor-based triplet to deepen the response before transplantation. Every other change of regimen, that is, a change after SD or PD to the preceding regimen or more than 6 months after the start of a regimen to which the patient had responded, was regarded as the start of a new line of therapy, irrespective of whether it was made for progression, toxicity or another reason. The 6-month window was a study-specific definition: neither the IMWG guidance nor the more recent EBMT framework specifies a time interval for this distinction, and both would generally regard an unplanned substitution of drugs for inadequate response as the start of a new line [17,18]. The window was chosen to approximate the usual duration of pre-transplant induction (about four to six cycles) and was applied only to patients who had responded to the regimen being changed, so that early switches made to deepen a response before planned transplantation were not counted as treatment failure. Because this rule is more lenient than the IMWG guidance, the stricter definition, in which every change of regimen other than ASCT and maintenance was counted as an event, was analyzed as TTNT (Section 2.3). First-line induction regimens were grouped as VCD, VAD, bortezomib–dexamethasone (VD), VRD and other regimens (bortezomib–melphalan–prednisone, melphalan–prednisone with or without thalidomide, bortezomib–doxorubicin–dexamethasone, daratumumab–VCD, lenalidomide–dexamethasone and dexamethasone–cyclophosphamide–etoposide–cisplatin). Response was assessed according to IMWG criteria [19] and recorded as the best response to the first induction regimen; response was not centrally reassessed, and the date on which the best response was first documented was not recorded in the database. For analysis, responses were grouped as CR/VGPR, PR/MR and SD/PD; MR was grouped with PR because both indicate a measurable but less than very good partial response and the MR group was small (*n* = 13).

The first transplant was classified with the same line-of-therapy rules: as first-line consolidation when it followed the first induction regimen, either directly or after an induction modification for suboptimal response, and as later-line transplantation when it followed the start of a new line of therapy, either after SD or PD to the preceding regimen or after a response. For each transplant, the time from diagnosis, the regimen given immediately before transplantation, the best response to that regimen (disease status at transplantation) and post-transplant maintenance were recorded. Transplant eligibility was determined by the treating hematologists on the basis of age, Eastern Cooperative Oncology Group (ECOG) performance status, organ function (cardiac, pulmonary, renal and hepatic) and response to induction therapy.

### 2.3. Study Endpoints

Overall survival (OS) was measured from the date of diagnosis to death from any cause. Vital status and dates of death were obtained for all patients from the national Death Notification System (Ölüm Bildirim Sistemi); patients alive at the administrative cut-off were therefore censored on 31 December 2025. Because exact radiological or clinical progression dates were not available retrospectively and the reason for each change of therapy was not recorded, progression-free survival could not be determined reliably. The treatment-based endpoint was therefore time to next line of therapy or death (TNLT), measured from diagnosis to the start of the second line of therapy, as defined in Section 2.2, or to death from any cause, whichever occurred first; patients alive who had not started a second line of therapy were censored at their last clinic visit. Induction modifications for suboptimal response, consolidation with ASCT and post-transplant maintenance were part of the first line and were therefore not counted as events. TNLT is not a measure of documented progression: a new line may have been started for toxicity or other reasons, and progression in a patient who received no further therapy contributes an event only at death. Time to next treatment or death (TTNT), in which every change of regimen other than ASCT and post-transplant maintenance, including induction modifications, was counted as an event, was analyzed as a secondary endpoint. TNLT and TTNT therefore differ only in the handling of induction modifications: TTNT does not rely on the rule that classified early changes after a response as part of the first line, and it was used to assess the sensitivity of the results to that rule. For eight patients whose recorded first-treatment date preceded the recorded diagnosis date, the earlier date was used as the index date. Two patients with internally inconsistent dates were excluded from the TNLT and TTNT analyses.

### 2.4. Statistical Analysis

Categorical variables are presented as numbers and percentages of patients with available data, with the number of patients with missing data reported for each variable, and continuous variables as medians with interquartile ranges (IQR). OS, TNLT and TTNT were estimated with the Kaplan–Meier method and compared with the log-rank test. Median follow-up was estimated with the reverse Kaplan–Meier method [20].

Multivariable Cox proportional hazards models were fitted with a prespecified set of clinically relevant covariates rather than by univariable selection: age (continuous, per 10 years), sex, ISS stage, LDH above the upper limit of normal, period of diagnosis (≤2014, 2015–2019, 2020–2024) and first-line induction regimen (model 2); model 1 omitted the induction regimen. ASCT was entered as a time-dependent covariate in a counting-process formulation to avoid immortal-time bias [21]. For each outcome, both models were fitted to the same complete-case sample, which provided 155 deaths (about 13 events per estimated parameter) for OS and 211 events for TNLT, and nested models were compared with the likelihood-ratio test and the Akaike information criterion (AIC). The proportional hazards assumption was checked with scaled Schoenfeld residuals, non-linearity of age was examined with natural cubic splines with three degrees of freedom, and influential observations were examined with scaled dfbeta residuals. Because the proportional hazards assumption was not met for transplantation and induction regimen, their associations were also estimated for prespecified follow-up intervals (OS: 0–24, 24–60 and >60 months; TNLT: 0–12, 12–36 and >36 months) by including interactions between these covariates and the follow-up interval in the counting-process model; the interval-specific model was compared with the proportional hazards model with the likelihood-ratio test, and models with interactions with log time were fitted as a sensitivity analysis [22]. As a summary measure that does not require proportional hazards, the restricted mean survival time (RMST) up to 60 months for OS and 36 months for TNLT was compared between induction regimens from diagnosis, and between patients transplanted or not transplanted within 12 months of diagnosis in a 12-month landmark analysis (up to 48 and 24 months after the landmark, respectively), by regression on pseudo-observations adjusted for the model covariates [23,24]. The association between period of diagnosis and induction regimen was quantified with Cramér’s V and collinearity with generalized variance inflation factors (GVIF) [25]; in a further sensitivity analysis, missing ISS and LDH values were multiply imputed by chained equations (30 imputations) [26].

Because response to first-line therapy is known only after treatment has been given, its association with outcome was assessed in landmark analyses [27] restricted to patients alive (OS) or alive without a second line of therapy (TNLT) 6 months after diagnosis, with time measured from the landmark and adjustment for age, ISS and period of diagnosis; 12-month landmark analyses were performed as a sensitivity analysis. Patients with SD or PD as best response who had not started a second line of therapy by the landmark remained at risk for TNLT and were included. Because the date of best response was not recorded, two further sensitivity analyses used only information available at the landmark: one was restricted to patients whose first induction regimen had ended (that is, a subsequent regimen or transplantation had started) before the landmark, and in the other, patients still receiving their first regimen at the landmark were analyzed as a separate group. Consequently, the primary landmark analyses classified patients by their best response to the first regimen, which, for patients still receiving that regimen at the landmark (109 of 212 patients in the 6-month and 53 of 189 in the 12-month OS analysis), may have been documented after the landmark; only these two sensitivity analyses were restricted to response information available at the landmark. Laboratory parameters (immunoglobulins, serum free light chains, NLR and PLR) measured at diagnosis were analyzed as continuous, log2-transformed covariates in Cox models for OS, unadjusted and adjusted for age and ISS, with Harrell’s C-index as the measure of discrimination; data-derived cut-offs were not used.

Sensitivity analyses used R-ISS instead of ISS and LDH, with and without the modified R-ISS assignments; excluded patients diagnosed before 2010; restricted the time-dependent transplant covariate to first-line transplants; assessed FISH-defined high-risk cytogenetics in FISH-tested patients; and used a delayed-entry (left-truncated) model in which each patient entered the risk set at the start of first-line treatment at our center, which corresponded to the first visit. To assess robustness to confounding by treatment era and patient characteristics, model 2 was also stratified by period of diagnosis and fitted within each period, restricted to patients aged ≤70 and ≤65 years, and further adjusted for renal impairment and the number of comorbidities (0, 1 or ≥2). Patients with and without FISH results were compared with the Mann–Whitney U and chi-square tests. A two-sided *p* < 0.05 was considered significant. Analyses were performed in R version 4.3.3 (R Foundation for Statistical Computing, Vienna, Austria) with the survival package (version 3.5-8). The interval-specific and log-time models, RMST, collinearity diagnostics, multiple imputation and response-timing sensitivity analyses were performed in Python 3.13 (Python Software Foundation, Wilmington, DE, USA) with the lifelines (version 0.30; https://lifelines.readthedocs.io, accessed on 23 October 2026) and statsmodels (version 0.15; https://www.statsmodels.org, accessed on 23 October 2026) packages; the primary multivariable models were reproduced identically in both environments.

## 3. Results

### 3.1. Patient Characteristics

The study included 254 patients. The median age at diagnosis was 68 years (range, 37–96), 154 patients (60.6%) were aged 65 years or older and 141 (55.7%) were men (Table 1). ISS stage III was present in 95 of 241 patients (39.4%) and LDH was elevated in 60 of 241 (24.9%). FISH results were available for 81 patients (31.9%); any abnormality was detected in 20 (24.7%) and high-risk cytogenetics in 9 (11.1%); because t(14; 16) was not part of the institutional FISH panel, some high-risk patients may have been classified as standard risk. Compared with untested patients, FISH-tested patients were younger (median 66 vs. 69 years, *p* = 0.017), less often had ISS stage III (27.2% vs. 42.2%, *p* = 0.030) and were more often diagnosed after 2014 (*p* < 0.001), whereas OS did not differ between the two groups (log-rank *p* = 0.814). R-ISS could be assigned to 166 patients (stage I, 7; stage II, 128; stage III, 31). Of these assignments, 141 did not require cytogenetic data (ISS II, *n* = 102; ISS I with elevated LDH, *n* = 9; ISS III with elevated LDH, *n* = 30), two were based on high-risk FISH, and 23 (all 7 R-ISS I and 16 R-ISS II derived from ISS III) assumed the absence of the untested t(14; 16) and are therefore modified R-ISS assignments; 88 patients could not be staged (Supplementary Table S11).

### 3.2. Treatment Patterns and Transplantation

First-line induction changed markedly over the study period (Table 2; *p* < 0.001). VAD was the most frequent regimen in patients diagnosed until 2014 (48 of 74, 64.9%), VCD in 2015–2019 (48 of 109, 44.0%) and VRD in 2020–2024 (32 of 71, 45.1%).

Seventy-nine patients (31.1%) underwent stem cell transplantation (78 autologous and one allogeneic; Table 3). Fifty-eight transplants were first-line consolidation, 36 directly after the first induction regimen and 22 after an induction modification for suboptimal response, and 21 were performed in a later line, 12 after a new line started after SD or PD to the first regimen and nine after a new line started after a response. The median time from diagnosis to transplantation was 8.7 months (IQR 6.3–13.4) overall and 11.0 months in the later-line group. Before transplantation, 58 patients (73.4%) were in VGPR or better and 21 (26.6%) in PR; no patient underwent transplantation with less than PR. The regimen given immediately before transplantation was VRD in 29 patients, VCD in 26, VAD in eight and carfilzomib–lenalidomide–dexamethasone in seven. Forty patients (50.6%) started lenalidomide-based or thalidomide maintenance within 12 months of transplantation, nine underwent a second autologous transplant and two a subsequent allogeneic transplant. Transplant recipients were younger than non-recipients (median age 61 vs. 72 years), and only 11 of the 112 patients aged ≥70 years underwent transplantation; the proportion transplanted increased from 25.7% (≤2014) to 29.4% (2015–2019) and 39.4% (2020–2024; Table 2).

### 3.3. Response to First-Line Induction

The best response to first-line induction was CR in 12 patients (4.9%), VGPR in 53 (21.5%), PR in 79 (32.0%), MR in 13 (5.3%), SD in 3 (1.2%) and PD in 87 (35.2%). Response differed by regimen (*p* < 0.001): CR/VGPR was achieved in 32 of 41 patients (78.0%) treated with VRD, 18 of 85 (21.2%) with VCD, 5 of 24 (20.8%) with VD and 5 of 83 (6.0%) with VAD, whereas SD/PD was most frequent after VAD (50.6%) and VD (50.0%). The associations of first-line response with OS and TNLT are reported in the landmark analyses in Section 3.4 and Section 3.5.

### 3.4. Overall Survival

The median follow-up estimated by the reverse Kaplan–Meier method was 101.2 months (95% confidence interval [CI], 90.8–126.7), and 173 patients (68.1%) died. The median OS was 54.6 months (95% CI, 40.7–64.0), with 2- and 5-year OS rates of 66.5% and 47.4% (Table 4, Figure 1). OS decreased with increasing stage in all three staging systems (Durie–Salmon, ISS and R-ISS; all *p* ≤ 0.002; Figure 2A). Median OS was 102.2, 77.7 and 30.1 months for ISS stages I, II and III, respectively. Shorter OS was also observed in patients aged 65 years or older (median 44.2 vs. 64.0 months, *p* = 0.034), in those with elevated LDH (37.5 vs. 63.1 months, *p* = 0.044) and in FISH-tested patients with high-risk cytogenetics (16.7 vs. 63.1 months, *p* = 0.015; t(14; 16) not tested). OS differed by first-line induction (*p* = 0.002), mainly owing to the short survival of patients treated with VD (median 16.1 months; Figure 3A), and did not differ by sex, lytic lesions, extramedullary plasmacytoma, light-chain type or period of diagnosis.

**Table 4:** Overall survival according to baseline and treatment characteristics.

Variable	*N*	Events	2-Year, % (95% CI)	5-Year, % (95% CI)	Median, Months (95% CI)	*p*
All patients	254	173	66.5 (60.9–72.6)	47.4 (41.5–54.2)	54.6 (40.7–64.0)	
**Age**						0.034
<65	100	61	72.9 (64.6–82.2)	55.2 (46.0–66.2)	64.0 (46.1–100.5)	
≥65	154	112	62.3 (55.1–70.5)	42.3 (35.0–51.2)	44.2 (30.1–60.8)	
**Sex**						0.974
Male	141	95	66.6 (59.3–74.9)	47.0 (39.2–56.3)	50.2 (38.0–72.6)	
Female	112	77	66.9 (58.7–76.2)	48.4 (39.7–58.9)	56.8 (34.5–78.7)	
**ISS stage**						<0.001
I	44	24	77.3 (65.8–90.7)	69.6 (57.1–85.0)	102.2 (79.6–NE)	
II	102	60	75.5 (67.6–84.3)	57.6 (48.5–68.3)	77.7 (55.0–104.3)	
III	95	76	57.8 (48.7–68.7)	30.0 (21.7–41.5)	30.1 (21.1–46.1)	
**R-ISS stage (recalculated)**						0.002
I	7	4	85.7 (63.3–100.0)	85.7 (63.3–100.0)	126.9 (61.2–NE)	
II	128	79	69.5 (62.0–78.0)	54.3 (46.1–63.9)	66.4 (47.1–98.1)	
III	31	28	57.9 (42.8–78.2)	23.8 (12.5–45.3)	30.1 (11.8–50.2)	
**Durie–Salmon stage**						<0.001
I	59	30	81.4 (72.0–91.9)	68.3 (57.1–81.6)	100.5 (73.3–NE)	
II	78	47	75.6 (66.7–85.8)	53.1 (42.7–65.9)	61.0 (44.2–126.6)	
III	105	84	56.1 (47.3–66.4)	34.8 (26.5–45.7)	32.6 (21.5–51.7)	
**LDH (>240 U/L)**						0.044
Normal	181	113	70.7 (64.4–77.6)	53.1 (46.1–61.1)	63.1 (47.1–79.6)	
High	60	47	59.9 (48.7–73.7)	37.6 (26.8–52.8)	37.5 (23.2–64.0)	
**High-risk cytogenetics (FISH-tested)**						0.015
Standard	72	42	66.7 (56.6–78.5)	56.2 (45.6–69.2)	63.1 (44.2–126.9)	
High-risk	9	8	44.4 (21.4–92.3)	11.1 (1.8–70.5)	16.7 (8.4–NE)	
**Lytic lesions**						0.694
Absent	62	42	67.7 (57.1–80.4)	52.4 (41.2–66.7)	64.0 (38.7–95.0)	
Present	189	128	66.1 (59.6–73.2)	45.3 (38.5–53.4)	47.1 (36.3–63.1)	
**Extramedullary plasmacytoma**						0.255
Absent	194	128	70.6 (64.4–77.3)	50.6 (43.8–58.4)	60.6 (47.1–73.3)	
Present	56	41	57.1 (45.5–71.7)	39.5 (28.3–55.0)	30.1 (21.5–79.3)	
**Light chain**						0.134
Kappa	156	98	69.8 (63.0–77.4)	48.0 (40.5–57.0)	57.5 (46.6–78.7)	
Lambda	90	68	61.1 (51.8–72.1)	48.3 (39.0–60.0)	44.8 (25.5–73.3)	
**Diagnosis period**						0.463
≤2014	74	60	68.9 (59.1–80.3)	52.7 (42.5–65.4)	62.0 (38.7–95.0)	
2015–2019	109	80	64.2 (55.8–73.9)	43.1 (34.8–53.5)	41.2 (31.9–64.0)	
2020–2024	71	33	67.5 (57.4–79.4)	46.6 (33.7–64.4)	56.8 (40.7–NE)	
**First-line induction**						0.002
VCD	85	54	74.1 (65.4–84.0)	51.9 (42.1–63.9)	63.8 (44.2–100.5)	
VAD	83	65	65.1 (55.6–76.2)	49.4 (39.7–61.4)	58.4 (33.7–86.0)	
VD	26	22	38.5 (23.7–62.5)	18.5 (8.1–42.2)	16.1 (5.9–44.1)	
VRD	41	16	78.0 (66.4–91.8)	56.6 (40.2–79.5)	138.3 (50.2–NE)	
Other	14	12	64.3 (43.5–95.0)	40.2 (20.7–78.0)	44.2 (23.6–NE)	

Kaplan–Meier estimates with log-rank test. NE: not estimable. Response and transplantation occur after diagnosis (see Table 5, Table 6 and Table 7). High-risk cytogenetics: del(17p) and/or t(4; 14); t(14; 16) was not part of the institutional FISH panel, so some high-risk patients may have been classified as standard risk. Abbreviations as in Table 1. Variable headings are shown in bold.

**Table 5:** Multivariable Cox regression analysis for overall survival and time to next line of therapy or death.

Variable	OS: HR (95% CI)	*p*	TNLT: HR (95% CI)	*p*
Age (per 10 years)	0.92 (0.78–1.08)	0.313	0.97 (0.85–1.12)	0.714
Female sex (vs. male)	1.05 (0.75–1.47)	0.779	0.76 (0.57–1.01)	0.062
ISS II (vs. I)	0.98 (0.60–1.59)	0.923	1.16 (0.76–1.75)	0.495
ISS III (vs. I)	1.98 (1.21–3.23)	0.006	1.74 (1.15–2.64)	0.009
LDH > 240 U/L	1.55 (1.06–2.27)	0.025	1.33 (0.95–1.86)	0.102
Diagnosis 2015–2019 (vs. ≤2014)	1.97 (1.24–3.12)	0.004	2.29 (1.51–3.47)	<0.001
Diagnosis 2020–2024 (vs. ≤2014)	2.17 (1.17–4.02)	0.013	2.80 (1.66–4.74)	<0.001
Induction VAD (vs. VCD)	1.43 (0.91–2.24)	0.125	2.14 (1.43–3.23)	<0.001
Induction VD (vs. VCD)	2.84 (1.59–5.05)	<0.001	1.23 (0.74–2.05)	0.427
Induction VRD (vs. VCD)	0.77 (0.41–1.45)	0.415	0.70 (0.44–1.11)	0.128
Induction other (vs. VCD)	1.77 (0.87–3.62)	0.116	1.21 (0.63–2.34)	0.571
ASCT (time-dependent)	0.36 (0.23–0.56)	<0.001	0.34 (0.21–0.53)	<0.001

Complete-case samples: OS, 235 patients and 155 deaths; TNLT, 233 patients and 211 events. ASCT was modeled as a time-dependent covariate. Because the proportional hazards assumption was not met for ASCT and induction regimen in the OS model (Supplementary Table S4), their HRs are averages over follow-up; interval-specific estimates are shown in Table 6. HR: hazard ratio; CI: confidence interval; other abbreviations as in Table 1.

**Table 6:** Interval-specific hazard ratios for transplantation and first-line induction regimen (model 2).

Variable	Overall Survival: HR (95% CI)			Time to Next Line of Therapy or Death: HR (95% CI)		
Months from Diagnosis	0–24	24–60	>60	0–12	12–36	>36
Events (after ASCT)	72 (4)	41 (8)	42 (22)	119 (6)	53 (12)	39 (22)
ASCT (time-dependent)	0.18 (0.06–0.52)	0.20 (0.09–0.45)	0.99 (0.50–1.96)	0.55 (0.23–1.32)	0.27 (0.14–0.54)	0.33 (0.16–0.71)
Induction VAD (vs. VCD)	1.65 (0.89–3.07)	1.44 (0.66–3.14)	0.96 (0.45–2.05)	2.49 (1.55–4.02)	1.52 (0.68–3.37)	1.67 (0.70–3.99)
Induction VD (vs. VCD)	4.16 (2.04–8.50)	3.90 (1.24–12.29)	0.25 (0.03–2.04)	1.38 (0.72–2.65)	1.50 (0.62–3.66)	0.62 (0.14–2.71)
Induction VRD (vs. VCD)	0.75 (0.32–1.76)	0.97 (0.34–2.74)	0.62 (0.13–2.87)	0.48 (0.25–0.92)	1.22 (0.57–2.61)	0.69 (0.24–2.03)
Induction other (vs. VCD)	1.19 (0.40–3.54)	2.61 (0.73–9.34)	4.18 (1.09–16.01)	0.90 (0.35–2.34)	1.84 (0.61–5.54)	1.27 (0.27–6.06)

Interval-specific HRs from model 2 (Table 5), in which ASCT (time-dependent) and induction regimen (reference VCD) were allowed a different HR in each follow-up interval and the other covariates (age, sex, ISS, LDH and period of diagnosis) a single HR; complete-case samples as in Table 5 (OS 235 patients/155 deaths; TNLT 233/211). Likelihood-ratio tests for time-varying effects: OS, ASCT *p* = 0.022, induction regimen *p* = 0.335, both *p* = 0.014; TNLT, ASCT *p* = 0.531, induction regimen *p* = 0.316, both *p* = 0.366. Events (after ASCT): events in each interval, with events in patients who had undergone transplantation in parentheses. Estimates with a continuous interaction with log time and RMST analyses are shown in Supplementary Tables S6 and S7. Abbreviations as in Table 1 and Table 5.

**Table 7:** Landmark analyses of response to first-line induction.

Analysis	Response Group (Best Response to First Induction)	Patients	Events	Adjusted HR (95% CI)	*p*
OS, 6-month landmark	CR/VGPR (CR 12, VGPR 51)	63	25	1.00 (reference)	
	PR/MR (PR 71, MR 13)	84	53	1.68 (1.03–2.75)	0.037
	SD/PD (SD 2, PD 63)	65	55	2.93 (1.77–4.85)	<0.001
TNLT, 6-month landmark	CR/VGPR (CR 12, VGPR 49)	61	49	1.00 (reference)	
	PR/MR (PR 70, MR 13)	83	75	1.32 (0.90–1.92)	0.152
	SD/PD (SD 2, PD 15)	17	17	3.55 (1.95–6.45)	<0.001
OS, 12-month landmark	CR/VGPR (CR 10, VGPR 50)	60	22	1.00 (reference)	
	PR/MR (PR 68, MR 11)	79	48	1.69 (1.00–2.84)	0.049
	SD/PD (SD 1, PD 49)	50	40	2.47 (1.42–4.30)	0.001
TNLT, 12-month landmark	CR/VGPR (CR 8, VGPR 38)	46	36	1.00 (reference)	
	PR/MR (PR 51, MR 5)	56	49	1.12 (0.71–1.77)	0.627
	SD/PD (SD 0, PD 7)	7	7	2.82 (1.18–6.78)	0.020

Patients alive (OS) or alive without a second line of therapy (TNLT) at the landmark with known first-line response and complete covariate data; time measured from the landmark. Adjusted for age, ISS and period of diagnosis. Patients with SD or PD as best response who had not started a second line of therapy by the landmark remained at risk for TNLT; all of them had an event during follow-up. The date of best response was not recorded; sensitivity analyses that used only information available at the landmark are shown in Supplementary Table S8. Abbreviations as in Table 1.

**Figure 1:**
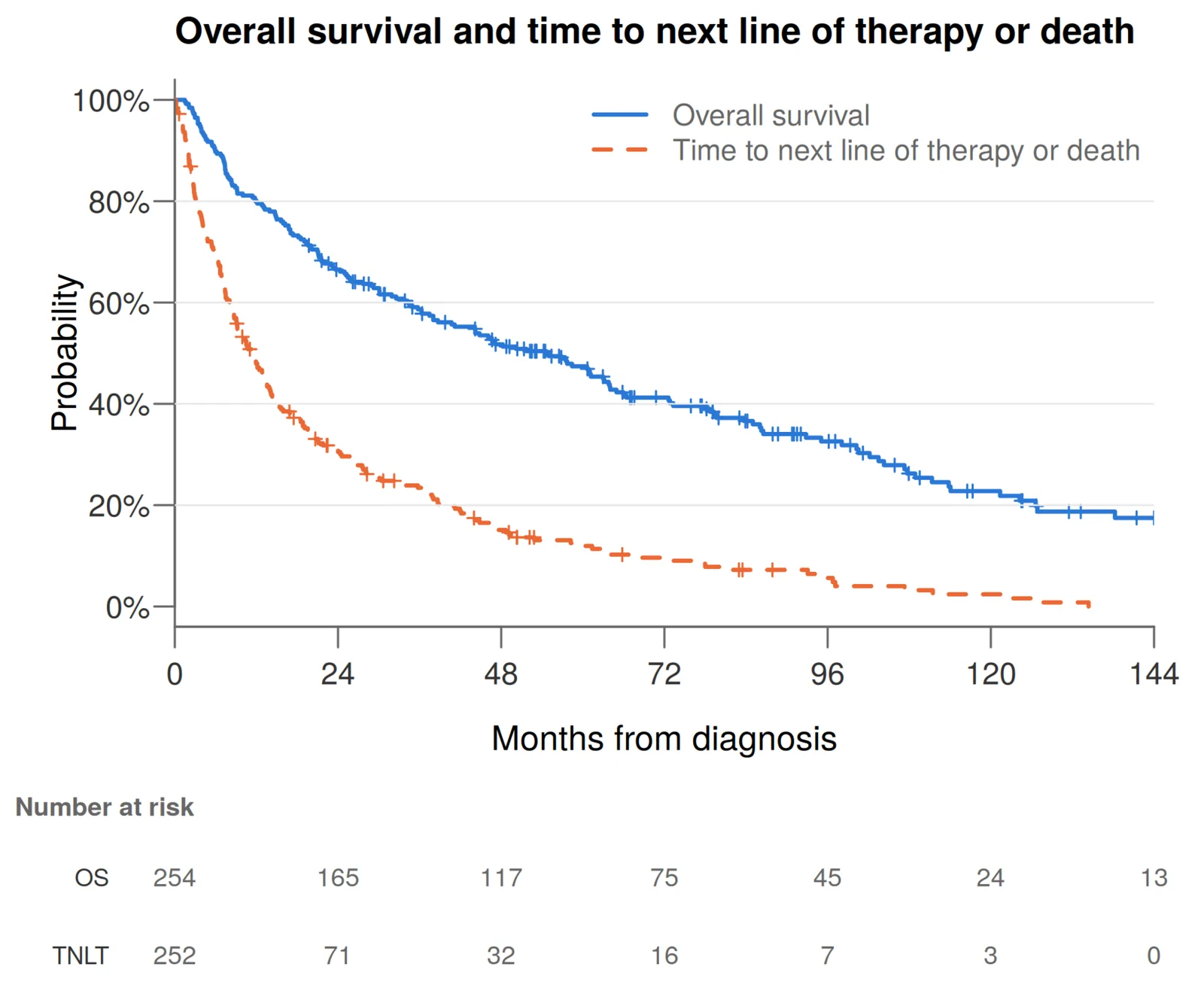
Overall survival and time to next line of therapy or death (TNLT) of the whole cohort. Tick marks indicate censored observations.

**Figure 2:**
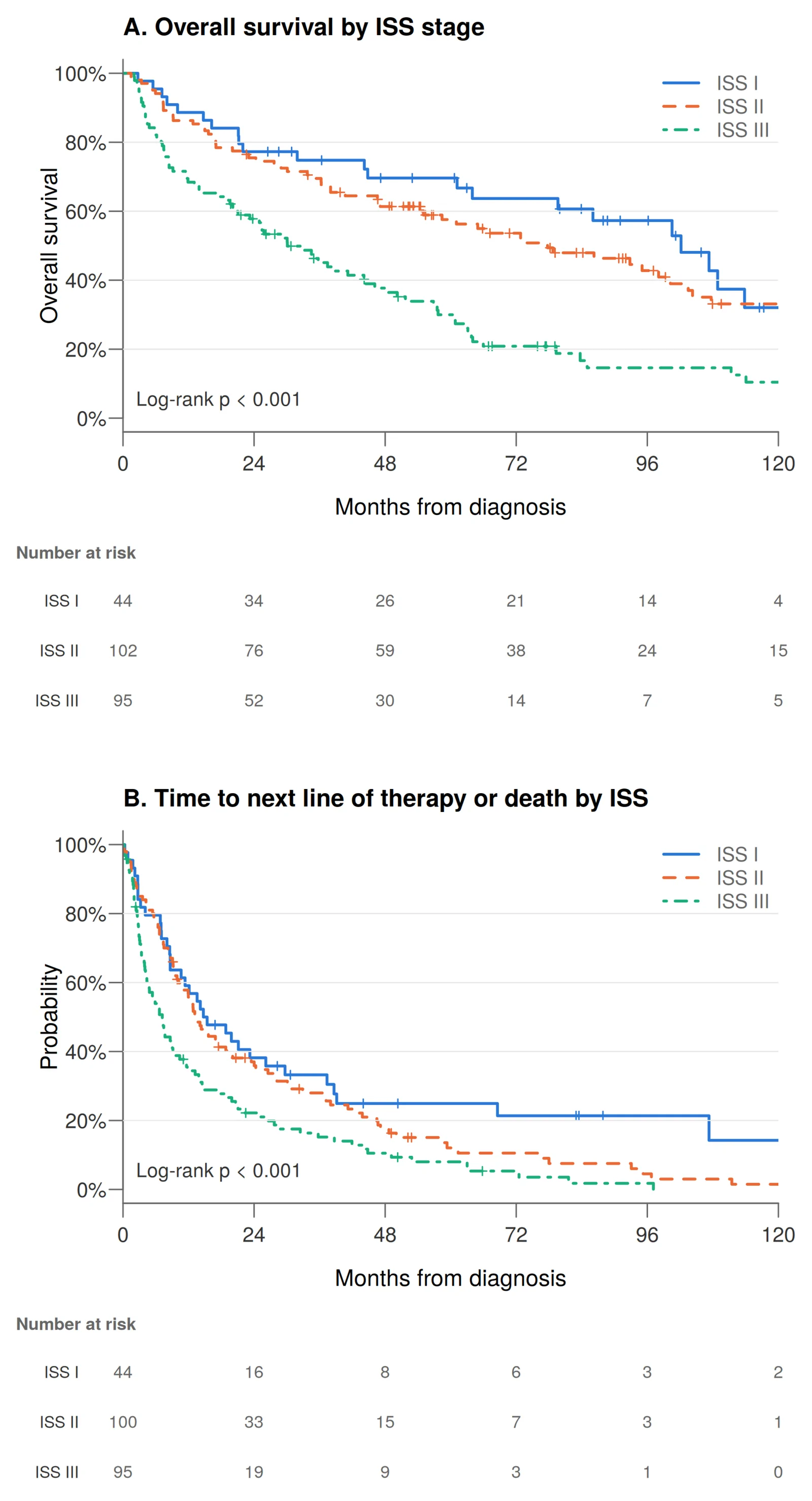
(**A**) Overall survival and (**B**) time to next line of therapy or death according to ISS stage.

**Figure 3:**
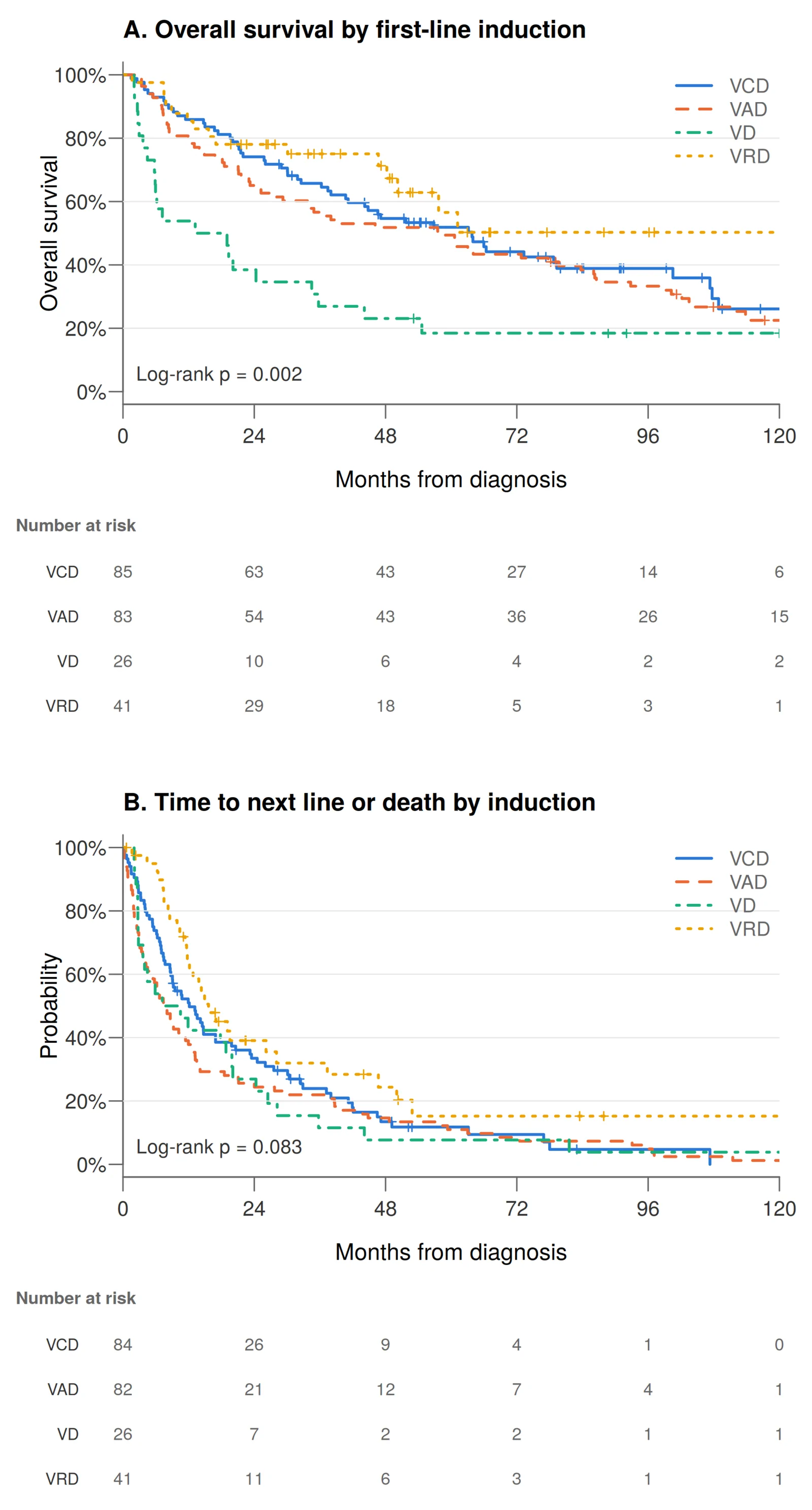
(**A**) Overall survival and (**B**) time to next line of therapy or death according to first-line induction regimen (VCD, VAD, VD and VRD; other regimens, *n* = 14). Differences are confounded by period of diagnosis and patient selection and should not be interpreted as treatment effects. The log-rank *p* values shown in Figure 3 refer to the four displayed induction groups (VCD, VAD, VD and VRD); the corresponding analyses in Table 4 and Table 8 additionally include the “Other” regimen group.

**Table 8:** Time to next line of therapy or death according to baseline and treatment characteristics.

Variable	*N*	Events	2-Year, % (95% CI)	5-Year, % (95% CI)	Median, Months (95% CI)	*p*
All patients	252	229	30.9 (25.6–37.3)	11.9 (8.3–17.1)	11.4 (9.1–13.9)	
**Age**						0.949
<65	98	82	31.8 (23.6–42.8)	17.3 (10.7–27.9)	9.0 (6.8–12.8)	
≥65	154	147	30.5 (24.0–38.8)	9.1 (5.4–15.4)	13.1 (9.9–15.4)	
**Sex**						0.026
Male	139	126	25.5 (19.0–34.1)	6.4 (3.0–13.5)	9.5 (7.5–14.0)	
Female	112	102	37.8 (29.8–48.0)	18.0 (11.9–27.1)	12.8 (10.4–19.1)	
**ISS stage**						<0.001
I	44	36	38.2 (26.1–55.8)	24.9 (14.6–42.6)	15.1 (10.6–37.4)	
II	100	91	37.0 (28.6–48.0)	12.1 (6.7–21.6)	13.2 (10.5–20.1)	
III	95	89	22.2 (15.1–32.6)	8.0 (3.9–16.4)	7.2 (4.8–10.4)	
**R-ISS stage (recalculated)**						0.005
I	7	6	57.1 (30.1–100.0)	28.6 (8.9–92.2)	26.2 (2.7–NE)	
II	126	113	34.4 (27.0–44.0)	12.1 (7.1–20.5)	12.4 (9.6–16.9)	
III	31	31	16.1 (7.2–36.0)	6.5 (1.7–24.7)	4.5 (2.8–10.4)	
**Durie–Salmon stage**						0.003
I	58	48	42.3 (31.2–57.3)	19.8 (11.3–34.7)	14.7 (10.6–27.6)	
II	77	70	33.8 (24.6–46.5)	14.5 (8.0–26.2)	14.0 (9.5–18.8)	
III	105	99	23.3 (16.4–33.1)	7.6 (3.7–15.4)	7.5 (6.3–11.4)	
**LDH (>240 U/L)**						0.359
Normal	179	161	31.4 (25.2–39.2)	12.6 (8.3–19.0)	12.8 (9.2–15.6)	
High	60	55	29.4 (19.8–43.7)	13.2 (6.4–26.9)	8.9 (6.3–12.1)	
**High-risk cytogenetics (FISH-tested)**						0.018
Standard	72	63	33.2 (23.8–46.4)	12.0 (6.0–23.7)	11.9 (7.5–20.1)	
High-risk	8	8	0.0 (NE)	0.0 (NE)	7.2 (3.6–NE)	
**Lytic lesions**						0.491
Absent	61	57	28.7 (19.2–42.8)	11.0 (5.2–23.0)	11.8 (8.5–19.1)	
Present	188	169	31.1 (25.1–38.6)	12.5 (8.3–18.9)	10.8 (8.6–14.0)	
**Extramedullary plasmacytoma**						0.223
Absent	192	174	31.6 (25.6–39.0)	12.4 (8.3–18.5)	11.4 (8.5–14.1)	
Present	56	51	31.0 (20.8–46.1)	11.8 (5.4–25.6)	11.9 (9.0–21.1)	
**Light chain**						0.082
Kappa	156	143	28.0 (21.7–36.2)	7.4 (3.9–14.0)	11.6 (8.7–14.2)	
Lambda	88	79	35.5 (26.7–47.2)	19.4 (12.5–30.2)	9.6 (7.5–20.6)	
**Diagnosis period**						0.101
≤2014	74	74	39.2 (29.5–52.1)	17.6 (10.7–28.8)	12.3 (8.6–26.0)	
2015–2019	107	100	25.5 (18.4–35.5)	7.2 (3.6–14.6)	9.9 (6.7–14.1)	
2020–2024	71	55	29.8 (20.5–43.2)	14.6 (7.4–28.6)	11.8 (9.1–18.8)	
**First-line induction**						0.156
VCD	84	74	33.5 (24.7–45.5)	11.8 (6.2–22.4)	12.1 (8.7–19.8)	
VAD	82	82	25.6 (17.7–37.0)	11.0 (5.9–20.3)	7.4 (5.5–12.0)	
VD	26	26	26.9 (14.3–50.7)	7.7 (2.0–29.1)	8.9 (3.6–24.3)	
VRD	41	30	39.1 (26.1–58.5)	15.2 (6.1–37.9)	15.6 (12.2–37.4)	
Other	14	13	35.7 (17.7–72.1)	14.3 (4.0–51.5)	17.1 (8.7–61.4)	

Kaplan–Meier estimates with log-rank test. Events: start of a second line of therapy (*n* = 179) or death before a second line (*n* = 50); 23 patients censored (Section 2.3; event composition in Supplementary Table S10). NE: not estimable. High-risk cytogenetics: del(17p) and/or t(4; 14); t(14; 16) was not part of the institutional FISH panel, so some high-risk patients may have been classified as standard risk. Abbreviations as in Table 1. Variable headings are shown in bold.

In the multivariable model for OS (235 patients, 155 deaths; Table 5), ISS stage III (HR 1.98, 95% CI 1.21–3.23), elevated LDH (HR 1.55, 1.06–2.27) and VD induction compared with VCD (HR 2.84, 1.59–5.05) were associated with shorter OS, and ASCT modeled as a time-dependent covariate was associated with longer OS (HR 0.36, 0.23–0.56). Age was not independently associated with OS (HR per 10 years 0.92, 0.78–1.08) and had a linear association with it (*p* for non-linearity 0.905). Adding the induction regimen improved model fit (likelihood-ratio test *p* = 0.004; Supplementary Table S2).

The proportional hazards assumption was not met for ASCT and induction regimen in the OS model (global test *p* = 0.002; Supplementary Table S4), so the HRs in Table 5 are averages over follow-up. When their associations were estimated for separate follow-up intervals (Table 6; likelihood-ratio test against the proportional hazards model, *p* = 0.014), ASCT was associated with lower mortality during the first 5 years after diagnosis (0–24 months: HR 0.18, 95% CI 0.06–0.52; 24–60 months: HR 0.20, 0.09–0.45) but not thereafter (HR 0.99, 0.50–1.96; interaction *p* = 0.022). No transplant recipient died during the first 16 months after diagnosis, so the earliest estimate rests on only four deaths after transplantation. VD induction was associated with higher mortality than VCD during the first 2 years (HR 4.16, 2.04–8.50) and from 2 to 5 years (HR 3.90, 1.24–12.29), but not after 5 years, when few VD-treated patients remained at risk (HR 0.25, 0.03–2.04); VAD and VRD did not differ significantly from VCD in any interval. Models with an interaction with log time showed the same pattern (Supplementary Table S6). In RMST analyses (Supplementary Table S7), patients treated with VD lived on average 17.7 months (95% CI 6.2–29.2) less than those treated with VCD during the first 5 years after diagnosis, and, in the 12-month landmark analysis, the 54 patients transplanted within 12 months of diagnosis lived on average 15.7 months (11.4–19.9) longer during the following 4 years than the 134 who were not, after adjustment for the model covariates.

Although crude OS did not differ by period of diagnosis in the whole cohort (log-rank *p* = 0.463), later periods were associated with higher adjusted hazards (2015–2019: HR 1.97; 2020–2024: HR 2.17 vs. ≤2014). Period of diagnosis and induction regimen were strongly associated (Cramér’s V 0.49), but the GVIF did not indicate numerical instability (GVIF^(1/(2·df)) ≤ 1.18 for all covariates; Supplementary Table S9). The 15 patients diagnosed until 2014 who were excluded from the complete-case sample because of missing ISS, LDH or treatment data had a median OS of 18.5 months, compared with 85.0 months for the 59 retained, so the reference period was enriched for long-term survivors (crude HR in the complete-case sample 1.55 for 2015–2019); with multiple imputation of missing ISS and LDH (248 patients), the HRs were attenuated (1.72 and 1.94) but not removed. Adding the induction regimen increased the period HRs (from 1.82 to 1.97 and from 1.54 to 2.17), indicating that, for a given regimen, patients diagnosed later had higher hazards.

Induction regimens overlapped mainly in 2015–2019, when VCD (*n* = 48), VAD (*n* = 31) and VD (*n* = 18) were used concurrently, whereas VAD and VD were rarely used after 2019 and VRD before 2015 (Table 2; Supplementary Table S12). With stratification by period of diagnosis, the OS estimates were similar (ASCT: HR 0.37; VD: HR 2.69), and within 2015–2019, ASCT (HR 0.31, 0.16–0.62) and VD (HR 2.82, 1.38–5.77) remained associated with OS. In patients aged ≤70 or ≤65 years, in whom transplantation was a realistic option, the ASCT HRs were 0.31 and 0.29, whereas the VD association was no longer evident (HR 1.44, 0.51–4.06, and 0.56, 0.06–4.76), consistent with VD having been used mainly in older patients. Further adjustment for renal impairment and the number of comorbidities (219 patients) did not change the ASCT (HR 0.32) or period estimates materially and attenuated the VD association (HR 2.43, 1.32–4.45); the higher adjusted hazards in later periods persisted in these analyses.

The OS estimates were also consistent in the other sensitivity analyses (Supplementary Table S3): R-ISS stage III was associated with shorter OS (HR 3.00, 1.01–8.86 vs. R-ISS I), and its HR vs. R-ISS II was 2.63 (1.64–4.20) when the 23 modified R-ISS assignments were excluded; restricting the transplant covariate to first-line transplants gave an HR of 0.39 (0.24–0.64); and results were similar when patients diagnosed before 2010 were excluded and in the delayed-entry model (ASCT HR 0.35). No single patient had a large influence on the estimates (maximum absolute scaled dfbeta 0.58), and excluding the five most influential patients did not change the direction of any of the main associations.

In the 6-month landmark analysis of first-line response (Table 7, Figure 4A), 212 patients were alive with known first-line response and complete covariate data (CR 12, VGPR 51, PR 71, MR 13, SD 2 and PD 63). Compared with CR/VGPR, SD/PD was associated with shorter OS (adjusted HR 2.93, 95% CI 1.77–4.85; PD alone, HR 2.83, 1.70–4.70) and PR/MR with a smaller increase in risk (HR 1.68, 1.03–2.75). The associations persisted at the 12-month landmark (SD/PD: HR 2.47, 1.42–4.30; PR/MR: HR 1.69, 1.00–2.84). The first induction regimen had ended before the landmark in 103 of the 212 patients at 6 months and in 136 of the 189 patients at 12 months. When only information available at the landmark was used, SD/PD remained associated with shorter OS than response (≥MR), with attenuated HRs (6-month landmark: 1.66, 1.00–2.75, in patients whose first regimen had ended, and 1.72, 1.05–2.82, when patients still receiving their first regimen were analyzed as a separate group, vs. 2.08, 1.44–3.02, in the corresponding primary analysis; Supplementary Table S8).

### 3.5. Time to Next Line of Therapy or Death

Among 252 patients evaluable for TNLT, 229 events occurred: 179 patients started a second line of therapy and 50 died before doing so. Of the 179 second lines, 84 were started after SD or PD to the preceding regimen, 93 after a recorded response (≥MR) to the preceding regimen (66 during first-line induction and 27 after transplantation or maintenance) and two after a regimen with unknown response; 58 induction modifications in 53 patients, 58 transplants and 31 maintenance therapies preceding the event were not counted as events (Supplementary Table S10). The median TNLT was 11.4 months (95% CI, 9.1–13.9), and the 2- and 5-year TNLT rates were 30.9% and 11.9% (Table 8, Figure 1). TNLT was shorter with higher stage (ISS *p* < 0.001, R-ISS *p* = 0.005, Durie–Salmon *p* = 0.003; Figure 2B), in men (median 9.5 vs. 12.8 months, *p* = 0.026) and in FISH-tested patients with high-risk cytogenetics (7.2 vs. 11.9 months, *p* = 0.018; t(14; 16) not tested). TNLT did not differ by age group (*p* = 0.949), LDH, lytic lesions, plasmacytoma, light-chain type, period of diagnosis or first-line induction (*p* = 0.156; Figure 3B). When induction modifications were also counted as events (TTNT), 236 events occurred and the median was 6.2 months (95% CI, 5.4–7.4); the directions of the multivariable associations were unchanged (Supplementary Table S5).

In the multivariable model for TNLT (233 patients, 211 events; Table 5), ISS stage III (HR 1.74, 1.15–2.64), VAD induction (HR 2.14, 1.43–3.23) and later periods of diagnosis (2015–2019: HR 2.29; 2020–2024: HR 2.80) were associated with shorter TNLT and ASCT with longer TNLT (HR 0.34, 0.21–0.53). Age was not associated with TNLT (HR per 10 years 0.97, 0.85–1.12; *p* for non-linearity 0.716), and adding the induction regimen improved model fit (*p* < 0.001; Supplementary Table S2). The association with ASCT did not vary significantly over time (Table 6; interaction *p* = 0.531; HR 0.27 and 0.33 after the first year), whereas VAD was associated with shorter TNLT (HR 2.49, 1.55–4.02) and VRD with longer TNLT (HR 0.48, 0.25–0.92) mainly during the first year. These estimates were similar with stratification by period of diagnosis (ASCT: HR 0.34; VAD: HR 2.20), within 2015–2019 (VAD: HR 2.84, 1.67–4.83), in patients aged ≤70 or ≤65 years (ASCT: HR 0.34 and 0.41) and after adjustment for renal impairment and comorbidity (ASCT: HR 0.31; Supplementary Table S12), and in the other sensitivity analyses (Supplementary Table S3): R-ISS stage III was associated with shorter TNLT (HR 5.25, 1.89–14.62 vs. R-ISS I; 2.61, 1.68–4.05 vs. R-ISS II without the modified assignments), restriction to first-line transplants gave an ASCT HR of 0.34 (0.21–0.54), and high-risk cytogenetics was associated with shorter TNLT in FISH-tested patients (HR 2.43, 1.11–5.33), although patients with t(14; 16) could not be identified.

In the 6-month landmark analysis (Table 7), 161 patients were alive without a second line of therapy, including 17 with SD or PD as best response to the first induction regimen (2 and 15, respectively) who had not yet started a further line. Compared with CR/VGPR, SD/PD was associated with shorter TNLT (HR 3.55, 1.95–6.45; all 17 patients had an event), whereas PR/MR was not (HR 1.32, 0.90–1.92; Figure 4B). Results were similar at the 12-month landmark (SD/PD: HR 2.82, 1.18–6.78; PR/MR: HR 1.12, 0.71–1.77).

### 3.6. Laboratory Parameters

None of the laboratory parameters measured at diagnosis was associated with OS when analyzed as a continuous variable (Supplementary Table S1). HRs per doubling ranged from 0.86 (PLR, *p* = 0.079) to 1.03 (NLR, *p* = 0.716), C-indices ranged from 0.49 to 0.53, and adjustment for age and ISS did not change these results. Serum free light chains were available for only 89 patients.

## 4. Discussion

In this single-center cohort of 254 patients followed for a median of more than 8 years, first-line treatment of MM changed from VAD to bortezomib-based triplets over two decades, and about one third of patients underwent transplantation, mostly as first-line consolidation. ISS stage, LDH, induction regimen and transplantation were associated with survival, with the associations of transplantation and VD induction with OS concentrated in the first 5 years after diagnosis, poor early response identified patients with a markedly worse prognosis, and routine laboratory parameters and inflammatory ratios added no prognostic information.

The median age of 68 years and the median OS of 54.6 months are consistent with descriptions of MM as a disease of older adults with heterogeneous outcomes [1]. All three staging systems stratified OS, confirming the relevance of the ISS and R-ISS outside the trial setting [9,10]. ISS stage III and elevated LDH remained associated with OS in the multivariable model, and R-ISS stage III with both outcomes in the subset in which it could be assigned, including when the modified assignments that assumed the absence of t(14; 16) were excluded. FISH was available for only one third of patients and did not include t(14; 16), so some high-risk patients may have been classified as standard risk, and high-risk cytogenetics could be evaluated only in a small, younger and lower-stage subgroup; even so, the nine high-risk patients had short OS and TNLT, in line with the prognostic weight of del(17p) and t(4; 14) [28]. Extramedullary plasmacytoma was not associated with outcome, possibly because of heterogeneous sites and the small number of affected patients [29].

The shift in induction therapy mirrors the introduction and reimbursement of novel agents during the study period. Response rates differed sharply by regimen, with CR/VGPR in 78% of VRD-treated patients but only 6% of VAD-treated patients, and more than one third of the cohort had SD/PD after the first regimen. This high rate of primary refractoriness, which was concentrated in the VAD and VD groups, largely explains the short median TNLT of 11.4 months. Because TNLT reflects the duration of first-line therapy in routine practice, including changes of therapy made for reasons other than progression, it cannot be compared directly with the progression-free survival reported in trials of contemporary regimens [5,30]. The associations between induction regimen and outcome should not be read as treatment effects: regimen choice was closely linked to period of diagnosis, access to drugs, age and fitness, and VD in particular was probably selected for frail patients or those with renal impairment; its excess mortality was confined to the first 5 years after diagnosis, was attenuated after adjustment for renal impairment, and was not evident in patients aged ≤70 years. The unexpected finding that later diagnosis was associated with higher adjusted hazards, despite no crude difference in the whole cohort, should not be read as a deterioration of outcomes. Period and regimen were strongly associated, although formal diagnostics did not indicate unstable estimates; part of the association reflected the exclusion of early-period patients with missing staging data and short survival from the complete-case sample, and the remainder is probably due to residual confounding, including changes over time in the patients selected for each regimen. It was unchanged in the delayed-entry analysis and attenuated, but not removed, by multiple imputation, and should be interpreted cautiously.

Transplantation was associated with markedly longer OS and TNLT, consistent with randomized and meta-analytic evidence supporting frontline ASCT [6,7] and with real-world data from Japan [13]. For OS, the association was confined to the first 5 years after diagnosis and was absent thereafter; this pattern, together with the absence of deaths among transplant recipients during the first 16 months, is what would be expected if recipients were selected from patients with a favorable early course. Because transplantation was modeled as a time-dependent covariate, the association is not explained by immortal-time bias [21]. For TNLT, the association also partly reflects the endpoint definition: transplantation and maintenance are part of the first line and require a response, so transplant recipients remain longer on first-line therapy by design, and their longer TNLT should not be interpreted as a longer progression-free interval. However, recipients were about ten years younger, had to have at least a PR, and were selected on performance status and organ function, so the magnitude of the association almost certainly overstates any causal benefit. The association was consistent within periods of diagnosis, in patients aged ≤70 years and after adjustment for renal impairment and comorbidity, but performance status was not available, and fitness cannot be fully captured by these variables. Age was not independently associated with OS once transplantation and the other covariates were included, which suggests that much of the crude age effect reflects transplant eligibility and fitness rather than chronological age itself; formal geriatric assessment, which was not available, would be needed to separate these factors [31].

In landmark analyses that avoid the guarantee-time bias inherent in comparing responders with non-responders from diagnosis [27], SD/PD after first-line induction was associated with a threefold higher hazard of death, and PR/MR with a smaller increase. This is consistent with the prognostic importance of response depth reported in pooled trial analyses [8]. Because the date of best response was not recorded, responses documented after the landmark may have been used for patients still receiving their first regimen; however, the association of SD/PD with shorter OS persisted, although attenuated, when only information available at the landmark was used. SD/PD was also associated with a shorter TNLT, but this association should be interpreted with caution, because the classification of treatment lines was itself response-dependent. Under our rules, any change of therapy after SD or PD started a new line and was therefore counted as an event, whereas a change within 6 months after at least MR was regarded as an induction modification and was not. The association of SD/PD with TNLT therefore partly reflects the endpoint definition rather than an independent prognostic effect; all 17 patients with SD/PD in the 6-month TNLT landmark analysis had an event. Conversely, because induction modifications were made mainly in patients in PR, not counting them as events may have prolonged TNLT in the PR/MR group and attenuated its difference from CR/VGPR, which may contribute to the absence of an association between depth of response and TNLT among responders in our cohort. The association of response with OS, which does not depend on the classification of treatment lines, is therefore the more reliable evidence of the prognostic value of early response in this cohort. Response, however, is an intermediate outcome that depends on the regimen received and on disease biology, and our data do not show that it mediates the effects of stage or treatment; it should be regarded as a post-baseline prognostic marker that identifies patients who may benefit from early change of therapy. Minimal residual disease, now the most powerful response-based predictor, was not available [32].

None of the laboratory parameters examined, including NLR and PLR, was associated with OS when analyzed as continuous variables. This differs from the meta-analytic association reported for NLR, although the same analysis found no association for PLR [12]. Single time-point measurement, heterogeneous cut-offs in the literature and limited statistical power may explain the discrepancy; our results argue against using these ratios in isolation for risk stratification.

This study has several limitations. First, its retrospective single-center design carries a risk of selection and information bias, and treatment allocation, including transplant eligibility, was not randomized, so residual confounding by indication cannot be excluded. Second, exact progression dates and the reasons for changes of therapy were not recorded, so progression-free survival could not be assessed; TNLT reflects treatment decisions as well as the course of the disease, a new line may have been started for toxicity or patient preference, and progression in patients who received no further therapy was captured only at death. The classification of early changes after a response as induction modifications relied on a prespecified, study-specific 6-month rule; counting these changes as events (TTNT) shortened the median but did not change the direction of the multivariable associations. Response was taken from the medical records, was not centrally reassessed and its date was not recorded, so the landmark analyses may include responses documented after the landmark. Third, FISH was available for only 81 patients, t(14; 16) was not part of the institutional FISH panel, so some high-risk patients may have been classified as standard risk, and R-ISS could be assigned to 166 patients, 23 of whom only under the assumption that the untested t(14; 16) was absent; FISH-tested patients were younger and had lower stage, so the cytogenetic analyses may not be representative. Fourth, the study spans more than two decades in which treatment changed substantially; newer agents, including anti-CD38 antibodies and carfilzomib, were used mainly at relapse, and period of diagnosis and regimen are strongly correlated. Fifth, the proportional hazards assumption was not met for transplantation and induction regimen in the OS model, so their single HRs represent averages over follow-up; the interval-specific estimates show that the associations of transplantation and VD induction with OS were concentrated in the first 5 years after diagnosis. Finally, missing data led to complete-case analyses of 233–235 patients in the multivariable models, which preferentially excluded early-period patients with short survival, and minimal residual disease, ECOG performance status and geriatric assessment were not available.

## 5. Conclusions

In this real-world cohort, first-line treatment of MM shifted from VAD to bortezomib-based triplets over two decades, and transplantation was used mainly as first-line consolidation. ISS stage and LDH were associated with survival; the associations of transplantation and VD induction with OS were concentrated in the first 5 years after diagnosis, and SD/PD after first-line induction identified patients with a poor prognosis and a short time to next line of therapy. Routine laboratory parameters and inflammatory ratios did not add prognostic information. Because these findings are observational, are subject to the selection of transplant recipients by age, fitness and response, and span changing treatment eras, they should be confirmed in larger multicenter studies that include complete cytogenetic profiling, minimal residual disease assessment and contemporary regimens.

## Figures and Tables

**Figure 4:**
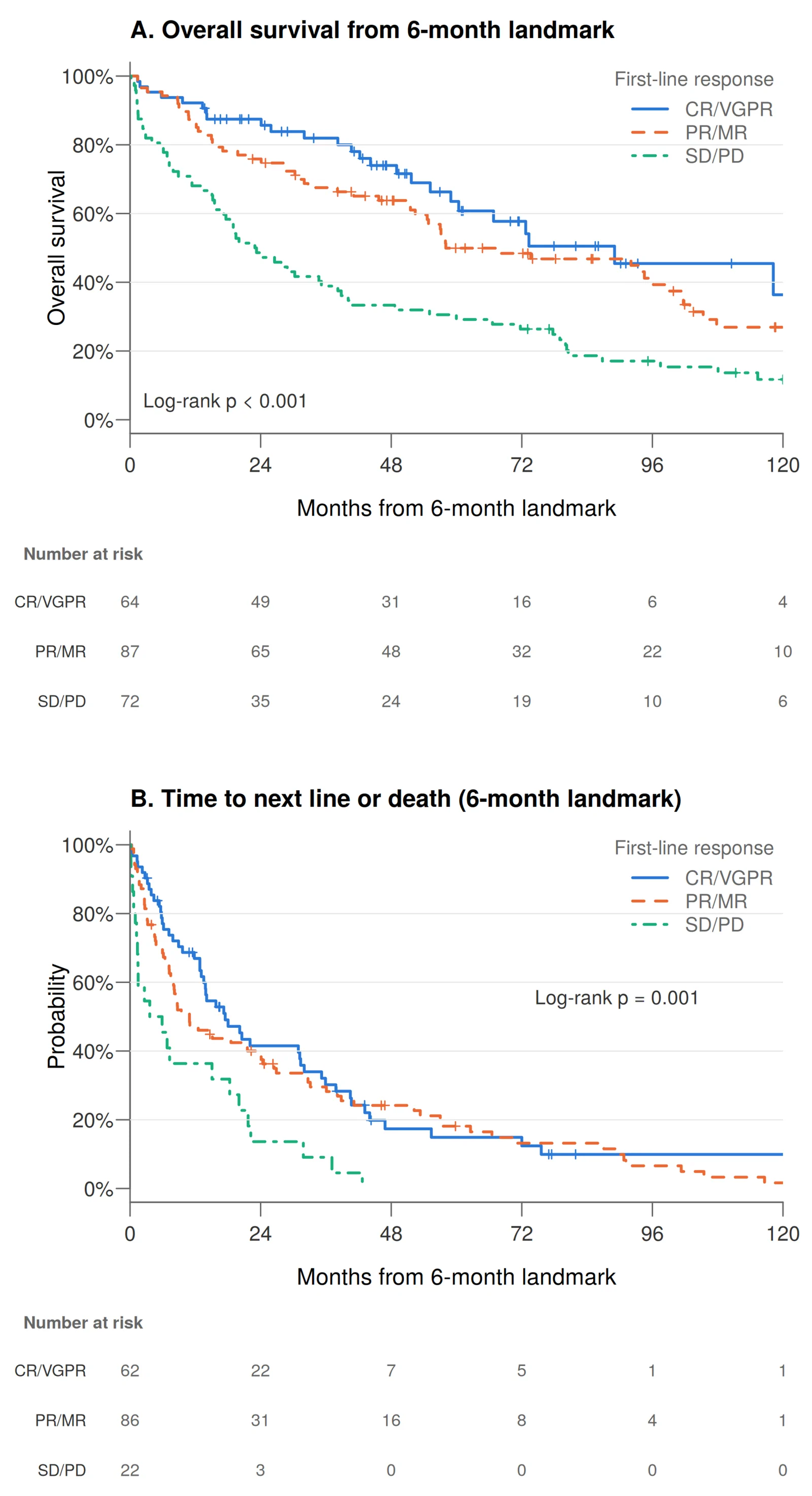
(**A**) Overall survival and (**B**) time to next line of therapy or death according to best response to first-line induction, 6-month landmark analysis. Kaplan–Meier curves include all patients with known first-line response at the landmark; adjusted HRs in Table 7 are from the complete-case models.

**Table 1:** Baseline characteristics, treatment and outcomes (*N* = 254).

Characteristic	*n* (%)
Age (years), median (range)	68 (37–96)
**Age group**	
<65 years	100 (39.4)
≥65 years	154 (60.6)
**Sex**	
Male	141 (55.7)
Female	112 (44.3)
Missing/not available	1
**Comorbidity (excluding myeloma-related conditions)**	
None	36 (15.6)
One	65 (28.1)
Two or more	130 (56.3)
Missing/not available	23
**Light chain**	
Kappa	156 (63.4)
Lambda	90 (36.6)
Missing/not available	8
**Durie–Salmon stage**	
I	59 (24.4)
II	78 (32.2)
III	105 (43.4)
Missing/not available	12
**ISS stage**	
I	44 (18.3)
II	102 (42.3)
III	95 (39.4)
Missing/not available	13
**LDH**	
Normal (≤240 U/L)	181 (75.1)
High (>240 U/L)	60 (24.9)
Missing/not available	13
**Interphase FISH**	
Performed	81 (31.9)
del(17p)/TP53 deletion, *n* (% of tested)	7 (8.6)
t(4; 14), *n* (% of tested)	3 (3.7)
t(11; 14), *n* (% of tested)	11 (13.6)
del(13q14), *n* (% of tested)	6 (7.4)
Any abnormality, *n* (% of tested)	20 (24.7)
High-risk [del(17p) and/or t(4; 14)], *n* (% of tested)	9 (11.1)
**R-ISS stage (recalculated; I and part of II modified, see footnote)**	
I	7 (4.2)
II	128 (77.1)
III	31 (18.7)
Not assessable (missing FISH and/or LDH)	88
**Lytic bone lesions**	
Present	189 (75.3)
Absent	62 (24.7)
Missing/not available	3
**Extramedullary plasmacytoma**	
Present	56 (22.4)
Absent	194 (77.6)
Missing/not available	4
Hemoglobin (g/dL), median (IQR)	10.0 (8.6–11.6); *n* = 244
Creatinine (mg/dL), median (IQR)	1.0 (0.8–1.7); *n* = 243
Creatinine ≥ 2 mg/dL, *n* (% of 243)	51 (21.0)
Calcium (mg/dL), median (IQR)	9.3 (8.7–10.2); *n* = 243
β2-microglobulin (mg/L), median (IQR)	4.7 (3.2–8.5); *n* = 205
Albumin (g/L), median (IQR)	32.0 (27.0–37.0); *n* = 244
**Period of diagnosis**	
≤2014	74 (29.1)
2015–2019	109 (42.9)
2020–2024	71 (28.0)
**First-line induction**	
VCD	85 (33.5)
VAD	83 (32.7)
VD	26 (10.2)
VRD	41 (16.1)
Other (VMP, MPT, MP, PAD, Dara-VCD, Rd, DCEP)	14 (5.5)
No treatment/unknown	5 (2.0)
**Best response to first-line induction (IMWG)**	
CR	12 (4.9)
VGPR	53 (21.5)
PR	79 (32.0)
MR	13 (5.3)
SD	3 (1.2)
PD	87 (35.2)
Missing/not available	7
**Stem cell transplantation (78 autologous, 1 allogeneic)**	
Any transplant	79 (31.1)
First line, after first induction	36 (14.2)
First line, after induction modification	22 (8.7)
Later line (after the start of a new line of therapy)	21 (8.3)
Maintenance started ≤12 months after transplant	40 (50.6% of transplanted)
Number of treatment lines, median (range)	2 (0–6)
Deaths	173 (68.1)
Start of a second line of therapy	180
Death before a second line of therapy	50

Percentages are calculated among patients with available data. R-ISS was calculated from ISS, LDH (upper limit of normal 240 U/L) and FISH [del(17p) and/or t(4; 14)]; t(14; 16) was not part of the institutional FISH panel, so some high-risk patients may have been classified as standard risk. Modified R-ISS: R-ISS I (*n* = 7) and R-ISS II derived from ISS III with normal LDH (*n* = 16) relied on standard-risk FISH without t(14; 16) testing. Comorbidities exclude conditions related to the plasma-cell disorder (plasmacytoma, amyloidosis, POEMS syndrome); ECOG performance status was not available. First-line transplantation: after the first induction regimen, directly or after an induction modification within 6 months for suboptimal response; later line: after the start of a new line of therapy, either after SD or PD to the preceding regimen or after a response. Of the 180 patients who started a second line of therapy, one was excluded from the TNLT analysis because of internally inconsistent dates; therefore, 179 such events are included in the TNLT analysis (Section 3.5). IQR: interquartile range; ISS: International Staging System; R-ISS: Revised ISS; LDH: lactate dehydrogenase; FISH: fluorescence in situ hybridization; VCD: bortezomib–cyclophosphamide–dexamethasone; VAD: vincristine–doxorubicin–dexamethasone; VD: bortezomib–dexamethasone; VRD: bortezomib–lenalidomide–dexamethasone; CR: complete response; VGPR: very good partial response; PR: partial response; MR: minimal response; SD: stable disease; PD: progressive disease. Variable headings are shown in bold.

**Table 2:** First-line induction regimen and transplantation according to period of diagnosis.

	≤2014 (*n* = 74)	2015–2019 (*n* = 109)	2020–2024 (*n* = 71)	All (*n* = 254)
**First-line induction**				
VCD	9 (12.2)	48 (44.0)	28 (39.4)	85 (33.5)
VAD	48 (64.9)	31 (28.4)	4 (5.6)	83 (32.7)
VD	6 (8.1)	18 (16.5)	2 (2.8)	26 (10.2)
VRD	1 (1.4)	8 (7.3)	32 (45.1)	41 (16.1)
Other	6 (8.1)	4 (3.7)	4 (5.6)	14 (5.5)
No treatment/unknown	4 (5.4)	0 (0.0)	1 (1.4)	5 (2.0)
**Stem cell transplantation**				
First line, after first induction	9 (12.2)	11 (10.1)	16 (22.5)	36 (14.2)
First line, after induction modification	5 (6.8)	10 (9.2)	7 (9.9)	22 (8.7)
Later line	5 (6.8)	11 (10.1)	5 (7.0)	21 (8.3)
No transplant	55 (74.3)	77 (70.6)	43 (60.6)	175 (68.9)

Values are *n* (% of the period column). Induction regimen differed across periods (chi-square test for VCD, VAD, VD and VRD, *p* < 0.001). Transplantation includes one allogeneic transplant. Variable headings are shown in bold.

**Table 3:** Characteristics of patients who underwent stem cell transplantation (*n* = 79).

	First Line, After First Induction	First Line, After Induction Modification	Later Line	All
Patients, *n*	36	22	21	79
Age at diagnosis, years, median (IQR)	61.0 (55.5–65.0)	64.5 (53.8–68.0)	61.0 (58.0–62.0)	61.0 (57.0–66.0)
Time from diagnosis to transplant, months, median (IQR)	8.1 (5.6–11.7)	9.4 (6.2–12.4)	11.0 (7.4–17.1)	8.7 (6.3–13.4)
Time from start of pre-transplant regimen, months, median (IQR)	7.0 (5.1–9.5)	5.3 (3.2–7.9)	5.9 (4.3–9.4)	6.5 (4.5–9.3)
**First-line induction**				
VCD	8 (22)	7 (32)	8 (38)	23 (29)
VRD	18 (50)	4 (18)	1 (5)	23 (29)
VAD	6 (17)	9 (41)	11 (52)	26 (33)
VD	3 (8)	1 (5)	1 (5)	5 (6)
Other	1 (3)	1 (5)	0 (0)	2 (3)
**Regimen immediately before transplant**				
VCD	8 (22)	10 (45)	8 (38)	26 (33)
VRD	18 (50)	5 (23)	6 (29)	29 (37)
VAD	6 (17)	2 (9)	0 (0)	8 (10)
KRD	0 (0)	4 (18)	3 (14)	7 (9)
VD	3 (8)	0 (0)	0 (0)	3 (4)
Other	1 (3)	1 (5)	4 (19)	6 (8)
**Disease status before transplant (response to preceding regimen)**				
CR	3 (8)	1 (5)	0 (0)	4 (5)
VGPR	20 (56)	18 (82)	16 (76)	54 (68)
PR	13 (36)	3 (14)	5 (24)	21 (27)
MR/SD/PD	0 (0)	0 (0)	0 (0)	0 (0)
**Post-transplant maintenance (started ≤12 months)**				
Lenalidomide	11 (31)	6 (27)	4 (19)	21 (27)
Lenalidomide–dexamethasone	6 (17)	7 (32)	5 (24)	18 (23)
Thalidomide	1 (3)	0 (0)	0 (0)	1 (1)
None	18 (50)	9 (41)	12 (57)	39 (49)
Second autologous transplant	3 (8)	2 (9)	4 (19)	9 (11)
Allogeneic transplant as first transplant	1 (3)	0 (0)	0 (0)	1 (1)
Subsequent allogeneic transplant	0 (0)	0 (0)	2 (10)	2 (3)

Values are *n* (%) unless otherwise stated. Induction modification: change of regimen within 6 months of its start in a patient with at least MR (in 22 patients: PR 17, VGPR 4, MR 1). Later line: transplantation after a new line of therapy started after SD or PD to the preceding regimen (*n* = 12) or after a response (*n* = 9). Disease status before transplantation is the best IMWG response to the regimen given immediately before transplantation. KRD: carfilzomib–lenalidomide–dexamethasone; other abbreviations as in Table 1. Variable headings are shown in bold.

## Data Availability

The data presented in this study are available on request from the corresponding author due to institutional data protection restrictions and patient privacy considerations.

## Supplementary Materials

The following supporting information can be downloaded at: https://www.mdpi.com/article/10.3390/jcm15218340/s1, Table S1: Laboratory parameters and overall survival (continuous Cox models); Table S2: Multivariable Cox regression without induction regimen (model 1); Table S3: Sensitivity analyses; Table S4: Proportional hazards diagnostics; Table S5: Time to next treatment or death (TTNT); Table S6: Time-varying hazard ratios with an interaction with log time; Table S7: Restricted mean survival time analyses; Table S8: Response categories in the landmark populations and response-timing sensitivity analyses; Table S9: Period of diagnosis, induction regimen and collinearity diagnostics; Table S10: Composition of TNLT events and treatment changes not counted as events; Table S11: Assignment of R-ISS stage and sensitivity analyses; Table S12: Treatment overlap across periods and robustness of the treatment-related estimates.
