# Supplementary material for: Treatment Patterns, Treatment Response, and Survival Outcomes in Patients with Multiple Myeloma: A Single-Center Real-World Study
Source: J Clin Med. 2026 Oct 26;15(21):8340. doi: 10.3390/jcm15218340 (PMC13764080; doi:10.3390/jcm15218340)
Supplement: Supplementary file 1 [file jcm-15-08340-s001.zip › jcm-4581888-supplementary.pdf]

Supplementary Materials

Treatment Patterns, Treatment Response, and Survival Outcomes in Patients with Multiple Myeloma: A Single-Center Real-World Study

Table S1. Laboratory parameters at diagnosis and overall survival (continuous Cox models).

Parameter	N	Deaths	Median (IQR)	HR per doubling (95% CI)	p	C-index	Adjusted HR (95% CI)	p
IgG	237	158	1450.0 (547.0–4090.0)	0.99 (0.91–1.07)	0.754	0.498	0.96 (0.89–1.04)	0.329
IgA	236	157	53.5 (26.0–154.0)	0.96 (0.90–1.02)	0.220	0.528	0.99 (0.93–1.06)	0.808
IgM	234	156	24.0 (22.0–38.0)	0.99 (0.84–1.16)	0.866	0.499	1.05 (0.90–1.22)	0.567
Serum free kappa	89	56	247.0 (31.0–1380.0)	1.02 (0.94–1.10)	0.637	0.491	1.01 (0.93–1.10)	0.744
Serum free lambda	89	56	44.0 (12.0–637.0)	1.00 (0.93–1.09)	0.905	0.514	0.99 (0.91–1.07)	0.731
NLR	244	163	2.1 (1.4–3.0)	1.03 (0.87–1.22)	0.716	0.506	1.04 (0.88–1.22)	0.683
PLR	244	163	125.6 (88.1–181.2)	0.86 (0.73–1.02)	0.079	0.517	0.88 (0.75–1.03)	0.112

Values were log2-transformed; HRs are per doubling of the parameter. Adjusted for age and ISS stage. Units: immunoglobulins, mg/dL; serum free light chains, mg/L. NLR: neutrophil-to-lymphocyte ratio; PLR: platelet-to-lymphocyte ratio; IQR: interquartile range.

Table S2. Multivariable Cox regression without induction regimen (model 1).

Variable	OS: HR (95% CI)	p	TNLT: HR (95% CI)	p
Age (per 10 years)	0.94 (0.80–1.10)	0.426	0.96 (0.84–1.10)	0.533
Female sex (vs. male)	1.08 (0.78–1.50)	0.643	0.76 (0.57–1.02)	0.064
ISS II (vs. I)	0.99 (0.61–1.60)	0.958	1.23 (0.81–1.85)	0.329
ISS III (vs. I)	2.18 (1.35–3.53)	0.002	1.86 (1.23–2.82)	0.003
LDH >240 U/L	1.47 (1.01–2.14)	0.044	1.34 (0.96–1.87)	0.090
Diagnosis 2015–2019 (vs. ≤2014)	1.82 (1.22–2.74)	0.004	1.45 (1.02–2.06)	0.040
Diagnosis 2020–2024 (vs. ≤2014)	1.54 (0.93–2.54)	0.094	1.33 (0.89–1.99)	0.170
ASCT (time-dependent)	0.41 (0.26–0.63)	<0.001	0.37 (0.24–0.58)	<0.001

Same complete-case samples as Table 5 (OS 235 patients/155 deaths; TNLT 233/211). AIC: OS 1448.4 (model 1) vs 1440.8 (model 2); TNLT 1882 vs 1870.2. Likelihood-ratio test for adding the induction regimen: OS p = 0.004, TNLT p < 0.001. ASCT modeled as a time-dependent covariate.

Table S3. Sensitivity analyses.

Model	Patients/events	Variable	HR (95% CI)	p
OS – R-ISS model	165/110	Age (per 10 years)	0.93 (0.78–1.12)	0.458
OS – R-ISS model	165/110	Female sex (vs. male)	1.08 (0.73–1.60)	0.706
OS – R-ISS model	165/110	R-ISS II (vs. I)	1.29 (0.46–3.61)	0.628
OS – R-ISS model	165/110	R-ISS III (vs. I)	3.00 (1.01–8.86)	0.047
OS – R-ISS model	165/110	Diagnosis 2015–2019 (vs. ≤2014)	1.73 (1.08–2.77)	0.024
OS – R-ISS model	165/110	Diagnosis 2020–2024 (vs. ≤2014)	1.60 (0.92–2.79)	0.099
OS – R-ISS model	165/110	ASCT (time-dependent)	0.48 (0.29–0.80)	0.005
OS – diagnosed 2010 or later	223/147	Age (per 10 years)	0.93 (0.78–1.10)	0.383
OS – diagnosed 2010 or later	223/147	Female sex (vs. male)	1.09 (0.77–1.55)	0.629
OS – diagnosed 2010 or later	223/147	ISS II (vs. I)	0.95 (0.57–1.60)	0.859
OS – diagnosed 2010 or later	223/147	ISS III (vs. I)	1.84 (1.09–3.08)	0.022
OS – diagnosed 2010 or later	223/147	LDH >240 U/L	1.57 (1.05–2.33)	0.026
OS – diagnosed 2010 or later	223/147	Diagnosis 2015–2019 (vs. ≤2014)	1.75 (1.09–2.82)	0.021
OS – diagnosed 2010 or later	223/147	Diagnosis 2020–2024 (vs. ≤2014)	1.96 (1.05–3.66)	0.035
OS – diagnosed 2010 or later	223/147	Induction VAD (vs. VCD)	1.51 (0.96–2.38)	0.074
OS – diagnosed 2010 or later	223/147	Induction VD (vs. VCD)	2.87 (1.61–5.13)	<0.001
OS – diagnosed 2010 or later	223/147	Induction VRD (vs. VCD)	0.75 (0.40–1.42)	0.382
OS – diagnosed 2010 or later	223/147	Induction other (vs. VCD)	1.57 (0.74–3.33)	0.235
OS – diagnosed 2010 or later	223/147	ASCT (time-dependent)	0.37 (0.23–0.60)	<0.001
OS – FISH-tested subset	81/50	Age (per 10 years)	1.34 (0.95–1.88)	0.095
OS – FISH-tested subset	81/50	ISS II (vs. I)	0.87 (0.37–2.06)	0.751
OS – FISH-tested subset	81/50	ISS III (vs. I)	2.83 (1.15–6.94)	0.023
OS – FISH-tested subset	81/50	High-risk FISH (vs. standard)	1.99 (0.89–4.46)	0.095
TNLT – R-ISS model	163/149	Age (per 10 years)	0.91 (0.77–1.06)	0.217
TNLT – R-ISS model	163/149	Female sex (vs. male)	0.76 (0.53–1.07)	0.111
TNLT – R-ISS model	163/149	R-ISS II (vs. I)	2.25 (0.87–5.83)	0.094
TNLT – R-ISS model	163/149	R-ISS III (vs. I)	5.25 (1.89–14.62)	0.001
TNLT – R-ISS model	163/149	Diagnosis 2015–2019 (vs. ≤2014)	1.84 (1.21–2.79)	0.005
TNLT – R-ISS model	163/149	Diagnosis 2020–2024 (vs. ≤2014)	1.41 (0.88–2.25)	0.149
TNLT – R-ISS model	163/149	ASCT (time-dependent)	0.38 (0.23–0.64)	<0.001
TNLT – diagnosed 2010 or later	221/199	Age (per 10 years)	0.98 (0.85–1.14)	0.831
TNLT – diagnosed 2010 or later	221/199	Female sex (vs. male)	0.82 (0.61–1.11)	0.198
TNLT – diagnosed 2010 or later	221/199	ISS II (vs. I)	1.12 (0.73–1.73)	0.609
TNLT – diagnosed 2010 or later	221/199	ISS III (vs. I)	1.63 (1.05–2.51)	0.029
TNLT – diagnosed 2010 or later	221/199	LDH >240 U/L	1.42 (1.00–2.02)	0.048
TNLT – diagnosed 2010 or later	221/199	Diagnosis 2015–2019 (vs. ≤2014)	1.96 (1.28–3.02)	0.002
TNLT – diagnosed 2010 or later	221/199	Diagnosis 2020–2024 (vs. ≤2014)	2.34 (1.36–4.03)	0.002
TNLT – diagnosed 2010 or later	221/199	Induction VAD (vs. VCD)	2.11 (1.39–3.19)	<0.001
TNLT – diagnosed 2010 or later	221/199	Induction VD (vs. VCD)	1.20 (0.71–2.02)	0.492
TNLT – diagnosed 2010 or later	221/199	Induction VRD (vs. VCD)	0.67 (0.42–1.07)	0.095
TNLT – diagnosed 2010 or later	221/199	Induction other (vs. VCD)	1.05 (0.53–2.09)	0.890
TNLT – diagnosed 2010 or later	221/199	ASCT (time-dependent)	0.39 (0.24–0.63)	<0.001

Model	Patients/events	Variable	HR (95% CI)	p
TNLT – FISH-tested subset	80/71	Age (per 10 years)	1.24 (0.95–1.62)	0.109
TNLT – FISH-tested subset	80/71	ISS II (vs. I)	1.32 (0.65–2.69)	0.449
TNLT – FISH-tested subset	80/71	ISS III (vs. I)	1.98 (0.92–4.29)	0.082
TNLT – FISH-tested subset	80/71	High-risk FISH (vs. standard)	2.43 (1.11–5.33)	0.026
OS – first-line transplant only	235/155	ASCT (time-dependent)	0.39 (0.24–0.64)	<0.001
TNLT – first-line transplant only	233/211	ASCT (time-dependent)	0.34 (0.21–0.54)	<0.001
OS – delayed entry	235/155	Age (per 10 years)	0.92 (0.78–1.08)	0.324
OS – delayed entry	235/155	Female sex (vs. male)	1.04 (0.74–1.45)	0.839
OS – delayed entry	235/155	ISS II (vs. I)	0.96 (0.59–1.56)	0.879
OS – delayed entry	235/155	ISS III (vs. I)	1.93 (1.18–3.15)	0.009
OS – delayed entry	235/155	LDH >240 U/L	1.55 (1.05–2.27)	0.026
OS – delayed entry	235/155	Diagnosis 2015–2019 (vs. ≤2014)	1.94 (1.22–3.07)	0.005
OS – delayed entry	235/155	Diagnosis 2020–2024 (vs. ≤2014)	2.10 (1.13–3.89)	0.018
OS – delayed entry	235/155	Induction VAD (vs. VCD)	1.42 (0.90–2.23)	0.130
OS – delayed entry	235/155	Induction VD (vs. VCD)	2.79 (1.57–4.97)	<0.001
OS – delayed entry	235/155	Induction VRD (vs. VCD)	0.79 (0.42–1.50)	0.474
OS – delayed entry	235/155	Induction other (vs. VCD)	1.78 (0.87–3.64)	0.112
OS – delayed entry	235/155	ASCT (time-dependent)	0.35 (0.22–0.56)	<0.001
TNLT – delayed entry	233/211	Age (per 10 years)	0.98 (0.85–1.13)	0.780
TNLT – delayed entry	233/211	Female sex (vs. male)	0.71 (0.53–0.95)	0.022
TNLT – delayed entry	233/211	ISS II (vs. I)	1.13 (0.75–1.70)	0.566
TNLT – delayed entry	233/211	ISS III (vs. I)	1.65 (1.08–2.50)	0.019
TNLT – delayed entry	233/211	LDH >240 U/L	1.33 (0.94–1.87)	0.106
TNLT – delayed entry	233/211	Diagnosis 2015–2019 (vs. ≤2014)	2.23 (1.47–3.38)	<0.001
TNLT – delayed entry	233/211	Diagnosis 2020–2024 (vs. ≤2014)	2.61 (1.55–4.40)	<0.001
TNLT – delayed entry	233/211	Induction VAD (vs. VCD)	2.21 (1.46–3.32)	<0.001
TNLT – delayed entry	233/211	Induction VD (vs. VCD)	1.18 (0.71–1.96)	0.520
TNLT – delayed entry	233/211	Induction VRD (vs. VCD)	0.75 (0.47–1.19)	0.221
TNLT – delayed entry	233/211	Induction other (vs. VCD)	1.31 (0.68–2.53)	0.423
TNLT – delayed entry	233/211	ASCT (time-dependent)	0.32 (0.20–0.51)	<0.001

R-ISS model: age, sex, R-ISS, period of diagnosis and time-dependent ASCT. Diagnosed 2010 or later: model 2 restricted to patients diagnosed from 2010 onward. FISH-tested subset: age, ISS and high-risk cytogenetics [del(17p) and/or t(4;14)]; t(14;16) was not part of the institutional FISH panel, so some high-risk patients may have been classified as standard risk. First-line transplant only: model 2 with the time-dependent covariate restricted to first-line transplants (58 patients; later-line transplants analyzed as no transplant) (only the transplant HR is shown). Delayed entry: model 2 with entry into the risk set at the start of first-line treatment at the center.

Table S4. Proportional hazards diagnostics for model 2 (scaled Schoenfeld residuals; p values).

Covariate	OS	TNLT
Age	0.063	0.100
Sex	0.176	0.765
ISS	0.563	0.206
LDH	0.313	0.684
Diagnosis period	0.781	0.114
Induction regimen	0.007	0.021
ASCT (time-dependent)	0.005	0.636
Global test	0.002	0.055

Test for non-linearity of age (natural cubic spline, 3 df): OS p = 0.905; TNLT p = 0.716. Because the assumption was not met for ASCT and induction regimen in the OS model, interval-specific HRs are shown in Table 6 and HRs from models with an interaction with log time in Table S6.

Table S5. Time to next treatment or death (TTNT; secondary endpoint), model 2.

Every regimen change other than transplantation and post-transplant maintenance was counted as an event: 236 events in 252 patients; median 6.2 (5.4–7.4) months; 2-year 22.0%, 5-year 7.5%. TTNT differs from TNLT (Table 5) only in that induction modifications are also counted as events.

Variable	HR (95% CI)	p
Age (per 10 years)	0.98 (0.85–1.12)	0.752
Female sex (vs. male)	0.84 (0.63–1.13)	0.245
ISS II (vs. I)	1.05 (0.70–1.58)	0.820
ISS III (vs. I)	1.46 (0.96–2.22)	0.076
LDH >240 U/L	1.15 (0.83–1.59)	0.411
Diagnosis 2015–2019 (vs. ≤2014)	2.79 (1.85–4.22)	<0.001
Diagnosis 2020–2024 (vs. ≤2014)	4.90 (2.84–8.48)	<0.001
Induction VAD (vs. VCD)	4.15 (2.71–6.35)	<0.001
Induction VD (vs. VCD)	1.35 (0.80–2.26)	0.260
Induction VRD (vs. VCD)	0.48 (0.31–0.76)	0.002
Induction other (vs. VCD)	1.36 (0.72–2.55)	0.342
ASCT (time-dependent)	0.26 (0.15–0.47)	<0.001

233 patients, 218 events. Abbreviations as in the main text.

Table S6. Time-varying hazard ratios for transplantation and induction regimen estimated with an interaction with log time (model 2).

Variable	p (interaction)	12 months	24 months	36 months	60 months	96 months
**Overall survival (235 patients, 155 deaths)**						
ASCT (time-dependent)	0.002	0.08 (0.03–0.27)	0.18 (0.09–0.39)	0.29 (0.16–0.51)	0.52 (0.31–0.86)	0.88 (0.45–1.72)
Induction VAD (vs. VCD)	0.265	1.66 (0.99–2.81)	1.44 (0.92–2.25)	1.32 (0.83–2.11)	1.19 (0.69–2.06)	1.08 (0.56–2.09)
Induction VD (vs. VCD)	0.004	3.06 (1.63–5.75)	1.84 (0.92–3.67)	1.36 (0.61–3.03)	0.93 (0.35–2.49)	0.66 (0.20–2.13)
Induction VRD (vs. VCD)	0.776	0.75 (0.37–1.52)	0.79 (0.42–1.48)	0.82 (0.41–1.63)	0.86 (0.36–2.02)	0.89 (0.31–2.60)
Induction other (vs. VCD)	0.097	1.25 (0.46–3.39)	2.01 (0.96–4.20)	2.66 (1.25–5.66)	3.78 (1.45–9.84)	5.22 (1.51–18.04)
**Time to next line of therapy or death (233 patients, 211 events)**						
ASCT (time-dependent)	0.693	0.37 (0.20–0.67)	0.34 (0.21–0.54)	0.33 (0.19–0.55)	0.31 (0.15–0.62)	0.29 (0.12–0.73)
Induction VAD (vs. VCD)	0.076	1.97 (1.29–3.00)	1.66 (1.01–2.75)	1.51 (0.85–2.67)	1.34 (0.68–2.62)	1.19 (0.55–2.59)
Induction VD (vs. VCD)	0.453	1.21 (0.70–2.06)	1.09 (0.57–2.11)	1.03 (0.48–2.21)	0.96 (0.38–2.38)	0.90 (0.31–2.59)
Induction VRD (vs. VCD)	0.073	0.68 (0.43–1.09)	0.89 (0.53–1.50)	1.03 (0.56–1.92)	1.25 (0.58–2.71)	1.49 (0.58–3.79)
Induction other (vs. VCD)	0.552	1.19 (0.61–2.32)	1.34 (0.60–2.99)	1.44 (0.56–3.73)	1.58 (0.49–5.13)	1.71 (0.42–7.03)

Model 2 (Table 5) with additional interactions between ASCT or induction regimen and the natural logarithm of time since diagnosis (episode splitting at each event time); the other covariates were modeled with a single HR. HRs (95% CI) are shown at selected times since diagnosis; p is the Wald test for the interaction with log time. Estimates at 12 months for ASCT rest on very few deaths after transplantation (no transplant recipient died during the first 16 months after diagnosis). The interval-specific estimates are shown in Table 6.

Table S7. Restricted mean survival time (RMST) analyses.

Group	Patients	RMST, months (crude)	Adjusted difference, months (95% CI)	p
**A. Induction regimen, overall survival up to 60 months from diagnosis (235 patients)**				
VCD	82	43.5	Reference	
VAD	78	40.3	3.0 (−3.9 to 10.0)	0.395
VD	23	23.0	−17.7 (−29.2 to −6.2)	0.002
VRD	40	47.6	11.6 (3.3 to 19.9)	0.006
Other regimens	12	39.1	−2.3 (−13.9 to 9.4)	0.702
**B. Induction regimen, time to next line of therapy or death up to 36 months from diagnosis (233 patients)**				
VCD	81	17.2	Reference	
VAD	77	13.3	−2.8 (−6.6 to 1.0)	0.147
VD	23	13.8	−1.9 (−8.3 to 4.6)	0.569
VRD	40	20.6	6.4 (1.1 to 11.8)	0.019
Other regimens	12	18.3	0.2 (−6.9 to 7.2)	0.963
**C. ASCT within 12 months, 12-month landmark, overall survival up to 48 months after the landmark**				
No ASCT by 12 months	134	34.7	Reference	
ASCT by 12 months	54	45.1	15.7 (11.4 to 19.9)	<0.001
**D. ASCT within 12 months, 12-month landmark, time to next line of therapy or death up to 24 months after the landmark**				
No ASCT by 12 months	76	14.0	Reference	
ASCT by 12 months	33	20.7	8.7 (5.2 to 12.1)	<0.001

RMST was estimated with the Kaplan–Meier method (crude) and compared by linear regression on jackknife pseudo-observations with robust standard errors, adjusted for age, sex, ISS, LDH, period of diagnosis and (panels C and D) induction regimen. Panels A and B are not adjusted for transplantation, which occurs after baseline, so the differences include any association mediated by transplantation (23 of 41 VRD-treated patients underwent transplantation). Panels C and D: patients alive (C) or alive without a second line of therapy (D) 12 months after diagnosis; transplantation after 12 months (25 and 19 patients, respectively) was analyzed in the "no ASCT by 12 months" group. Positive differences indicate longer survival time than the reference group.

Table S8. Response-timing sensitivity analyses for the landmark analyses.

Landmark / analysis	Patients/events	Comparison	HR (95% CI)	p
**6-month landmark, overall survival**				
Primary analysis	212/133	SD/PD vs. response (≥MR)	2.08 (1.44–3.02)	<0.001
Primary analysis, PD only (SD excluded)	210/131	PD vs. CR/VGPR	2.83 (1.70–4.70)	<0.001
First regimen ended before the landmark	103/68	SD/PD vs. response (≥MR)	1.66 (1.00–2.75)	0.050
Information available at the landmark	212/133	SD/PD vs. response (≥MR)	1.72 (1.05–2.82)	0.032
	212/133	Still on first regimen vs. response (≥MR)	1.32 (0.85–2.06)	0.218
**6-month landmark, time to next line of therapy or death**				
Primary analysis	161/141	SD/PD vs. CR/VGPR	3.55 (1.95–6.45)	<0.001
	161/141	PR/MR vs. CR/VGPR	1.32 (0.90–1.92)	0.152
First regimen ended before the landmark	53/44	PR/MR vs. CR/VGPR	1.66 (0.69–4.01)	0.255
Information available at the landmark	164/143	PR/MR vs. CR/VGPR	1.56 (0.69–3.55)	0.289
	164/143	Still on first regimen vs. CR/VGPR	2.45 (1.11–5.40)	0.026
**12-month landmark, overall survival**				
Primary analysis	189/110	SD/PD vs. response (≥MR)	1.74 (1.15–2.64)	0.009

Landmark / analysis	Patients/events	Comparison	HR (95% CI)	p
Primary analysis, PD only (SD excluded)	188/109	PD vs. CR/VGPR	2.37 (1.36–4.15)	0.002
First regimen ended before the landmark	136/78	SD/PD vs. response (≥MR)	1.66 (1.04–2.68)	0.036
Information available at the landmark	189/110	SD/PD vs. response (≥MR)	1.64 (1.03–2.63)	0.037
	189/110	Still on first regimen vs. response (≥MR)	1.35 (0.85–2.15)	0.207
**12-month landmark, time to next line of therapy or death**				
Primary analysis	109/92	SD/PD vs. CR/VGPR	2.82 (1.18–6.78)	0.020
	109/92	PR/MR vs. CR/VGPR	1.12 (0.71–1.77)	0.627
First regimen ended before the landmark	58/45	PR/MR vs. CR/VGPR	0.95 (0.43–2.13)	0.909
Information available at the landmark	111/93	PR/MR vs. CR/VGPR	1.18 (0.56–2.46)	0.665
	111/93	Still on first regimen vs. CR/VGPR	1.80 (0.86–3.78)	0.118

All models adjusted for age, ISS and period of diagnosis; patients with complete covariate data. Response categories in each landmark population are shown in Table 7. The date of best response was not recorded. "First regimen ended before the landmark": restricted to patients in whom a subsequent regimen or transplantation had started on or before the landmark (6 months: 103 of 212 patients for OS; 12 months: 136 of 189), so that the best response to the first regimen was necessarily established before the landmark. "Information available at the landmark": patients still receiving their first regimen (no subsequent therapy started) at the landmark were analyzed as a separate group, and only patients whose first regimen had ended were classified by response. Because few patients with CR/VGPR had ended their first regimen by 6 months (13 patients, 2 deaths), responders (≥MR) were combined as the reference group for the OS sensitivity analyses. In the 6-month OS landmark, the first regimen had ended before the landmark in 50 of 65 patients with SD/PD, 42 of 84 with PR/MR and 13 of 63 with CR/VGPR. For TNLT, patients with SD or PD as best response who had not started a second line of therapy by the landmark were included (Table 7); because any change of therapy after SD or PD starts a second line, none of them had ended their first regimen before the landmark, so they are not represented in the "first regimen ended" analyses and are included in the "still on first regimen" group of the "information available" analyses, together with patients whose response was not recorded.

Table S9. Period of diagnosis, induction regimen and collinearity diagnostics.

**A. Induction regimen by period of diagnosis, model 2 complete-case OS sample (n = 235); n (% of period)**						
**Period**	**VCD**	**VAD**	**VD**	**VRD**	**Other**	**Total**
≤2014	8 (13.6)	43 (72.9)	3 (5.1)	1 (1.7)	4 (6.8)	59
2015–2019	47 (43.5)	31 (28.7)	18 (16.7)	8 (7.4)	4 (3.7)	108
2020–2024	27 (39.7)	4 (5.9)	2 (2.9)	31 (45.6)	4 (5.9)	68
Cramér's V = 0.49 (chi-square p < 0.001); Cramér's V for the four main regimens (VCD, VAD, VD, VRD) = 0.50						

**B. Generalized variance inflation factors, GVIF^(1/(2·df))**			
**Covariate**	**df**	**Model 2**	**Model 1**
Age	1	1.18	1.12
Sex	1	1.02	1.01
ISS	2	1.03	1.02
LDH	1	1.05	1.04
Diagnosis period	2	1.17	1.02
Induction regimen	4	1.11	–
ASCT (ever)	1	1.14	1.12

**C. HRs (95% CI) for period of diagnosis (vs. ≤2014) with sequential adjustment**

Model	OS 2015–2019	OS 2020–2024	TNLT 2015–2019	TNLT 2020–2024
Crude	1.55 (1.05–2.27)	1.35 (0.82–2.21)	1.47 (1.06–2.06)	1.19 (0.81–1.75)
+ age, sex, ISS, LDH	1.83 (1.22–2.76)	1.54 (0.93–2.54)	1.45 (1.02–2.07)	1.26 (0.84–1.88)
+ ASCT (time-dependent) [model 1]	1.82 (1.22–2.74)	1.54 (0.93–2.54)	1.45 (1.02–2.06)	1.33 (0.89–1.99)
+ induction regimen [model 2]	1.97 (1.24–3.12)	2.17 (1.17–4.02)	2.29 (1.51–3.47)	2.80 (1.66–4.74)
Model 2 with multiple imputation (OS 248/168; TNLT 246/224)	1.71 (1.12–2.63)	1.94 (1.09–3.47)	2.16 (1.46–3.18)	2.65 (1.61–4.36)

**D. Overall survival of patients included in and excluded from the complete-case sample, by period**			
**Period**	**Included: n; median OS, months**	**Excluded: n; median OS, months**	**Log-rank p**
≤2014	59; 85.0	15; 18.5	<0.001
2015–2019	108; 41.2	1; 32.6	0.555
2020–2024	68; 56.8	3; 16.7	0.461

GVIF: generalized variance inflation factor (Fox and Monette), computed from the correlation matrix of the model covariates, with transplantation entered as ever/never; GVIF^(1/(2·df)) is comparable with the square root of the conventional variance inflation factor, and values below about 2 indicate no relevant collinearity. Panel C: crude and sequentially adjusted Cox models in the common complete-case samples (OS 235 patients/155 deaths; TNLT 233/211); the multiple-imputation model included all patients with a first-line regimen and known sex, with missing ISS (n = 11) and LDH (n = 11) imputed by chained logistic regression (30 imputations; predictors age, sex, period, regimen, albumin, creatinine, hemoglobin, vital status and the Nelson–Aalen cumulative hazard) and estimates combined with Rubin's rules. Panel D: patients were excluded from the complete-case sample because of missing ISS or LDH, missing sex or no first-line treatment.

Table S10. Composition of TNLT events and treatment changes not counted as a new line of therapy.

Category	n	Comment
**TNLT events (252 evaluable patients)**		
Start of a second line of therapy	179	
– After SD or PD to the preceding regimen (primary refractory disease)	84	Refractoriness documented by the recorded best response
– After a recorded response (≥MR) during first-line induction	66	Change >6 months after the start of the preceding regimen
– After a recorded response following ASCT or maintenance	27	Includes changes >12 months after transplantation
– After a regimen with unknown response	2	
Death before a second line of therapy	50	18 had PD as best response to first induction and received no further therapy
Censored at last visit	23	
**Treatment changes before the TNLT event that were not counted as a new line of therapy**		
Induction modification (change within 6 months after ≥MR)	58 (53 patients)	Part of the first line; counted as events in TTNT
Transplantation as first-line consolidation	58 (57 patients)	Part of the first line
Maintenance started ≤12 months after transplantation	31 (31 patients)	Part of the first line
**Comparison of endpoints (252 patients)**		
TNLT (start of a second line of therapy, or death): median, months	11.4	229 events
Time to next treatment or death (TTNT): median, months	6.2	236 events; every regimen change except ASCT and maintenance

Dates of progression documented according to IMWG criteria were not recorded, so progression-free survival could not be determined. Lines of therapy were classified from the recorded sequence, dates and best response of each regimen according to IMWG line-of-therapy principles (Section 2.2): a planned sequence of induction (including induction modification for

suboptimal response), transplantation and maintenance was regarded as one line, and every other change of regimen was counted as the start of a new line, irrespective of its reason. Because the reasons for changes of therapy were not recorded systematically, second lines started for toxicity or patient preference are included among the events, and TNLT is not a measure of documented progression. TNLT and TTNT differ only in the handling of induction modifications.

Table S11. Assignment of R-ISS stage according to available components, and sensitivity analyses.

Assigned R-ISS stage	Basis of assignment	n	Cytogenetic assumption
II	ISS II (irrespective of LDH and cytogenetics)	102	None
II	ISS I with elevated LDH	9	None
III	ISS III with elevated LDH	30	None
III	ISS III with normal LDH and high-risk FISH	1	None (high-risk abnormality detected)
II	ISS I with normal LDH and high-risk FISH	1	None (high-risk abnormality detected)
I	ISS I with normal LDH and standard-risk FISH	7	Absence of t(14;16) assumed (modified R-ISS)
II	ISS III with normal LDH and standard-risk FISH	16	Absence of t(14;16) assumed (modified R-ISS)
Not assessable	ISS I or III with normal or missing LDH and no FISH	75	Not staged; cytogenetics not presumed
Not assessable	ISS missing	13	
**Sensitivity analyses (age, sex, R-ISS, period of diagnosis and time-dependent ASCT)**			
OS	R-ISS III vs. II, all 166 assignments	165/110	2.32 (1.48–3.66)
OS	R-ISS III vs. II, modified assignments excluded	142/93	2.63 (1.64–4.20)
TNLT	R-ISS III vs. II, all 166 assignments	163/149	2.33 (1.52–3.57)
TNLT	R-ISS III vs. II, modified assignments excluded	140/129	2.61 (1.68–4.05)

R-ISS was calculated from ISS stage, LDH (upper limit of normal 240 U/L) and FISH-defined high-risk cytogenetics [del(17p) and/or t(4;14)]; t(14;16) was not part of the institutional FISH panel. Assignments marked "modified R-ISS" relied on standard-risk FISH and therefore on the assumption that t(14;16) was absent; untested patients whose stage depended on cytogenetics were not staged. In the sensitivity analyses, n/events refers to patients with complete covariate data; HRs (95% CI) compare R-ISS III with R-ISS II. Excluding the modified assignments removes all R-ISS I patients.

Table S12. Treatment overlap across periods of diagnosis and robustness of the treatment-related estimates.

**A. First-line induction regimen and transplantation by period of diagnosis: n (transplanted, n)**					
**Regimen**	**≤2014**	**2015–2019**	**2020–2024**	**All**	**Median age, years**
VCD	9 (2)	48 (11)	28 (10)	85 (23)	68
VAD	48 (15)	31 (11)	4 (0)	83 (26)	64
VD	6 (1)	18 (4)	2 (0)	26 (5)	74
VRD	1 (1)	8 (6)	32 (16)	41 (23)	64
Other regimens	6 (0)	4 (0)	4 (2)	14 (2)	79
Transplanted, % of period	25.7	29.4	39.4	31.1	

**B. Transplantation by age group**			
**Age group**	**Patients**	**Transplanted**	**% transplanted**
<60	56	29	51.8
60-64	44	24	54.5
65-69	42	15	35.7
≥70	112	11	9.8

C. Robustness of model 2 estimates, HR (95% CI)				
**Analysis (OS patients/deaths; TNLT patients/events)**	**ASCT, OS**	**ASCT, TNLT**	**VD vs. VCD, OS**	**VAD vs. VCD, TNLT**
Model 2 (Table 5) (235/155; 233/211)	0.36 (0.23–0.56)	0.34 (0.21–0.53)	2.84 (1.59–5.05)	2.14 (1.43–3.23)
Stratified by period of diagnosis (235/155; 233/211)	0.37 (0.23–0.58)	0.34 (0.21–0.54)	2.69 (1.51–4.80)	2.20 (1.45–3.32)
Diagnosed ≤2014 only (59/45; 59/59)	0.41 (0.18–0.94)	0.32 (0.13–0.80)	5.72 (1.11–29.39)	0.69 (0.25–1.89)
Diagnosed 2015–2019 only (108/79; 106/99)	0.31 (0.16–0.62)	0.38 (0.19–0.78)	2.82 (1.38–5.77)	2.84 (1.67–4.83)
Diagnosed 2020–2024 only (68/31; 68/53)	0.36 (0.12–1.07)	0.31 (0.12–0.79)	1.91 (0.21–17.06)	4.13 (1.05–16.26)
Age ≤70 years (147/96; 145/127)	0.31 (0.19–0.52)	0.34 (0.18–0.62)	1.44 (0.51–4.06)	2.47 (1.45–4.22)
Age ≤65 years (105/62; 103/85)	0.29 (0.16–0.55)	0.41 (0.19–0.90)	0.56 (0.06–4.76)	3.22 (1.70–6.10)
Adjusted for renal impairment and comorbidity (219/145; 217/197)	0.32 (0.19–0.52)	0.31 (0.19–0.52)	2.43 (1.32–4.45)	2.07 (1.34–3.18)

D. Period of diagnosis (vs. ≤2014), HR (95% CI)				
**Analysis**	**OS 2015–2019**	**OS 2020–2024**	**TNLT 2015–2019**	**TNLT 2020–2024**
Model 2 (Table 5)	1.97 (1.24–3.12)	2.17 (1.17–4.02)	2.29 (1.51–3.47)	2.80 (1.66–4.74)
Age ≤70 years	1.80 (1.01–3.21)	2.70 (1.24–5.90)	2.30 (1.36–3.89)	2.54 (1.28–5.07)
Age ≤65 years	2.32 (1.12–4.84)	3.66 (1.43–9.34)	2.24 (1.16–4.35)	2.39 (1.05–5.45)
Adjusted for renal impairment and comorbidity	1.80 (1.11–2.94)	1.87 (1.00–3.53)	2.26 (1.44–3.55)	2.86 (1.65–4.96)

Panel A: all 254 patients (5 without first-line treatment not shown). Panel C: model 2 covariates (age, sex, ISS, LDH, period of diagnosis, induction regimen and time-dependent ASCT); in the stratified model, period was used as a stratification factor, and within-period models omit period. Renal impairment: serum creatinine ≥2 mg/dL; comorbidity: number of comorbidity groups (0, 1, ≥2), excluding conditions related to the plasma-cell disorder. ECOG performance status was not available. Some within-period and age-restricted estimates are imprecise because of small numbers (for example, VD in 2020–2024, n = 2; VD in patients aged ≤65 years).
